# Unveiling the Crucial Roles of O_2_^•–^ and ATP in Hepatic Ischemia–Reperfusion Injury Using Dual-Color/Reversible Fluorescence Imaging

**DOI:** 10.1021/jacs.3c04303

**Published:** 2023-09-01

**Authors:** Jihong Liu, Wen Zhang, Xin Wang, Qi Ding, Chuanchen Wu, Wei Zhang, Luling Wu, Tony D. James, Ping Li, Bo Tang

**Affiliations:** † College of Chemistry, Chemical Engineering and Materials Science, Key Laboratory of Molecular and Nano Probes, Ministry of Education, Collaborative Innovation Center of Functionalized Probes for Chemical Imaging in Universities of Shandong, Institutes of Biomedical Sciences, Shandong Normal University, Jinan 250014, People’s Republic of China; ‡ Laoshan Laboratory, Qingdao 266237, People’s Republic of China; § Department of Chemistry, University of Bath, Bath BA2 7AY, U.K.; ∥ School of Chemistry and Chemical Engineering, Henan Normal University, Xinxiang 453007, People’s Republic of China

## Abstract

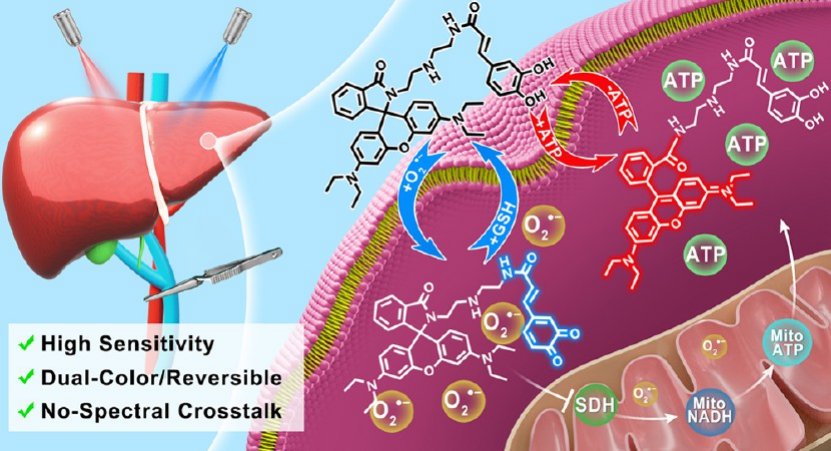

Hepatic ischemia–reperfusion injury (HIRI) is mainly responsible for morbidity or death due to graft rejection after liver transplantation. During HIRI, superoxide anion (O_2_^•^^–^) and adenosine-5′-triphosphate (ATP) have been identified as pivotal biomarkers associated with oxidative stress and energy metabolism, respectively. However, how the temporal and spatial fluctuations of O_2_^•^^–^ and ATP coordinate changes in HIRI and particularly how they synergistically regulate each other in the pathological mechanism of HIRI remains unclear. Herein, we rationally designed and successfully synthesized a dual-color and dual-reversible molecular fluorescent probe (UDP) for dynamic and simultaneous visualization of O_2_^•^^–^ and ATP in real-time, and uncovered their interrelationship and synergy in HIRI. UDP featured excellent sensitivity, selectivity, and reversibility in response to O_2_^•^^–^ and ATP, which rendered UDP suitable for detecting O_2_^•^^–^ and ATP and generating independent responses in the blue and red fluorescence channels without spectral crosstalk. Notably, in situ imaging with UDP revealed for the first time synchronous O_2_^•^^–^ bursts and ATP depletion in hepatocytes and mouse livers during the process of HIRI. Surprisingly, a slight increase in ATP was observed during reperfusion. More importantly, intracellular O_2_^•^^–^—succinate dehydrogenase (SDH)—mitochondrial (Mito) reduced nicotinamide adenine dinucleotide (NADH)—Mito ATP—intracellular ATP cascade signaling pathway in the HIRI process was unveiled which illustrated the correlation between O_2_^•^^–^ and ATP for the first time. This research confirms the potential of UDP for the dynamic monitoring of HIRI and provides a clear illustration of HIRI pathogenesis.

## Introduction

Hepatic ischemia–reperfusion injury (HIRI) is a severe but unavoidable complication of liver resection and transplantation surgery, which involves two interrelated stages of partial ischemic insult and subsequent inflammatory-mediated reperfusion injury.^1^ In clinical practice, HIRI can cause up to 10% of acute graft dysfunction during liver transplantation,^2^ increase the risk of postoperative mortality and morbidity, and even result in remote organ failure.^3^ Hence, accurate and real-time monitoring of the dynamic progression of HIRI is essential for timely intervention and treatment. Traditional methods for the diagnosis of liver injury rely on the determination of serum alanine aminotransferase (ALT) and aspartate aminotransferase (AST),^4^ which are nonspecific biomarkers of late-stage hepatocyte injury and are also influenced by other diseases^5^ and, therefore, fail to reflect changes of targets in the liver. Imaging diagnostic techniques for the assessment of liver injury including magnetic resonance imaging (MRI), computed tomography (CT), and ultrasonic (US) imaging are only suitable for evaluating histological pathologies and liver functional changes due to the inherent low sensitivity and specificity.^2^ Liver biopsy is an invasive method for clinical diagnosis of liver injury. However, it provides limited diagnostic accuracy due to observer variability and sampling bias,^6^ only provides static pathological status, and is unsuitable for the monitoring of the dynamic development of HIRI. Therefore, the development of a noninvasive and reliable method that enables real-time and dynamic monitoring of HIRI is needed, which will facilitate monitoring of disease progression, formulate treatment strategies, and improve surgery outcomes for HIRI.

In the progression of HIRI, enhanced oxidative stress and impairment of energy metabolism synergistically interact, collectively playing critical roles in liver injury.^7,8^ As a major reactive oxygen species (ROS), superoxide anion (O_2_^•^^–^) is produced excessively via an enzymatic reaction catalyzed by xanthine oxidase and nicotinamide adenine dinucleotide phosphate (NADPH) oxidase during HIRI,^4,9^ and triggers abnormal oxidative/nitrative stress, hepatocytes death, and further localized inflammation, ultimately resulting in liver dysfunction and failure.^10^ Meanwhile, as the ″molecular currency″ of energy transfer, adenosine-5′-triphosphate (ATP) depletion during ischemia is a critical hallmark in the process of HIRI.^11^ During HIRI, ATP depletion causes intracellular calcium ion overload, thereby leading to a cascade of intracellular pathological events such as mitochondrial permeability transition (MPT) pore opening, cytochrome C release, and eventual cell apoptosis.^7^ Therefore, O_2_^•^^–^ and ATP are potential biomarkers for the early detection of HIRI at the molecular level. While some progress has been made in the field of detecting HIRI using O_2_^•^^–^ or ATP as an oxidative stress marker or energy metabolism marker, respectively,^12−14^ how the temporal and spatial fluctuations of O_2_^•^^–^ and ATP coordinate changes in the whole process of HIRI and particularly how they synergistically regulate each other in the pathological mechanism of HIRI remain unclear, in a large part due to the lack of reliable methods for the simultaneous and in situ monitoring of O_2_^•^^–^ and ATP in vivo during HIRI. Colorimetry and high-performance liquid chromatography have been used to measure endogenous O_2_^•^^–^ or ATP levels in cell lysates and tissue homogenates during HIRI;^15−17^ unfortunately, the mechanisms for determining O_2_^•^^–^ or ATP are different, independent, and generally incompatible with the detection of O_2_^•^^–^ or ATP in living biological systems. Therefore, to meet this demand, a new method enabling not only real-time detection of O_2_^•^^–^ and ATP, but also elucidation of their interrelationship and synergy in HIRI is of great importance, and could hopefully realize the early detection of HIRI at the molecular level and decipher the potential etiology of HIRI.

Fluorescence imaging based on optical probes is emerging as an ideal approach to monitor and understand various biological and physiological processes intuitively and effectively due to excellent sensitivity, high spatial and temporal resolution, visualization of biological events at the molecular level, and noninvasive imaging.^18−24^ In particular, simultaneous imaging probes with multiple recognition sites have attracted more and more attention thanks to their inherent advantages of eliminating false signals and improving sensitivity and accuracy, which can be applied to understand the relationship between multiple bioactive molecules involved in disease progression and diagnose diseases precisely.^25,26^ Although a series of O_2_^•^^–^- or ATP-responsive fluorescence probes have been reported,^27−32^ none of them has enabled the simultaneous and in situ visualization of O_2_^•^^–^ and ATP in biological systems. If O_2_^•^^–^- or ATP-reactive fluorescent probes are used separately, the correlation between O_2_^•^^–^ and ATP remains elusive due to the limitations of nonquantitative uptake, different spatial distribution, and spectral crosstalk. In addition, the existing O_2_^•^^–^- or ATP-responsive fluorescent probes are not suitable for dynamic monitoring of O_2_^•^^–^ and ATP fluctuations in HIRI on account of their irreversible mechanisms. Therefore, we set out to develop a fluorescent probe capable of the simultaneous and reversible detection of O_2_^•^^–^ and ATP during HIRI, which can not only monitor temporal and spatial dynamics of O_2_^•^^–^ and ATP in the HIRI process but also decipher the correlation and regulatory mechanism between O_2_^•^^–^ and ATP in HIRI.

To meet the above requirements, rhodamine lactam skeleton combined with caffeic acid moiety was utilized to fabricate a dual-color and dual-reversible molecular platform (UDP) for the real-time detection and dynamic imaging of O_2_^•^^–^ and ATP in their independent blue and red fluorescence channels (O_2_^•^^–^: blue channel, λ_ex_ = 380 nm, λ_em_ = 470 nm; ATP: red channel, λ_ex_ = 520 nm, λ_em_ = 588 nm) without spectral crosstalk. UDP exhibited up to 26-fold and 204-fold fluorescence response upon activation by O_2_^•^^–^ and ATP, respectively. UDP also exhibited high selectivity and reversibility toward O_2_^•^^–^ and ATP. The synergistic effect between O_2_^•^^–^ and ATP in hepatocytes stimulated by 2-methylestradiol (2-ME, O_2_^•^^–^ promoter) and oligomycin A (Omy A, ATP inhibitor) was visualized. Meanwhile, real-time imaging of O_2_^•^^–^ and ATP dynamics in the process of HIRI in hepatocytes and mouse livers was realized using UDP, which was verified using commercial ROS and ATP assay kits. Notably, a combination of fluorescence imaging by UDP and biochemical data from commercial kits elucidated intracellular O_2_^•^^–^—succinate dehydrogenase (SDH)—mitochondrial (Mito) reduced nicotinamide adenine dinucleotide (NADH)—Mito ATP—intracellular ATP cascade molecular mechanisms in the process of HIRI for the first time.

## Results and Discussion

### Design and Synthesis of UDP

The rational design of a fluorescent probe capable of the simultaneous imaging and dynamic detection of O_2_^•^^–^ and ATP in vivo needs to satisfy three prerequisites. First, the response groups for O_2_^•^^–^ and ATP should be sufficiently sensitive and specific for the capture of O_2_^•^^–^ and ATP in complicated living systems. Second, the response to O_2_^•^^–^ and ATP needs to be reversible to enable dynamic monitoring of O_2_^•^^–^ and ATP. Lastly, the fluorescence emission peaks with O_2_^•^^–^- and ATP-relevant fluorophores should be well-separated to avoid crosstalk for the simultaneous detection of O_2_^•^^–^ and ATP, enabling the capture of authentic and reliable images of O_2_^•^^–^ and ATP in organisms.

**Figure 1:**
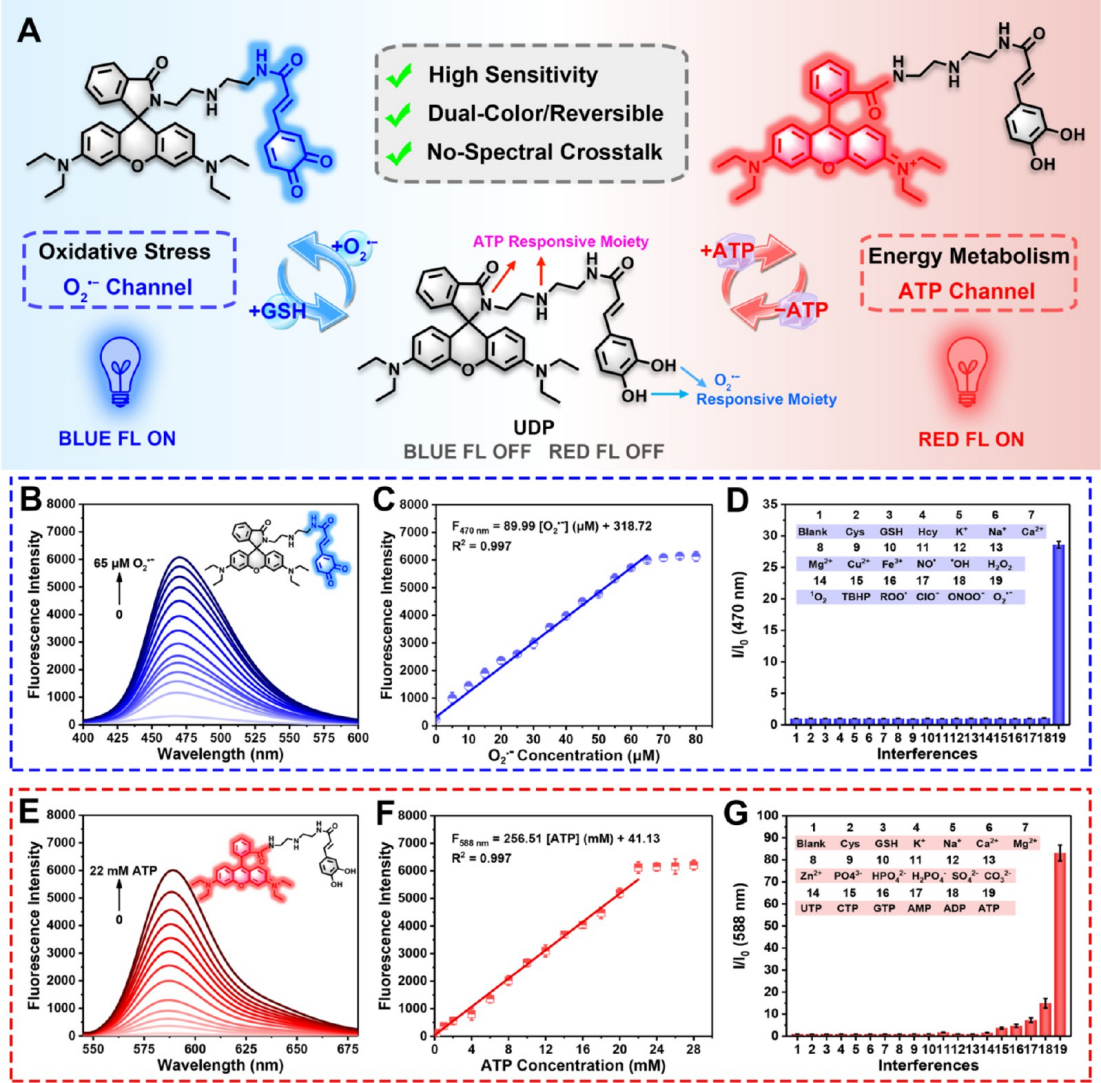
Structure and optical properties of UDP. (A) Luminescence reversible mechanisms of UDP in response to O_2_^•^^–^ and ATP. (B) Fluorescence spectra of UDP (25 μM) after incubation with different concentrations of O_2_^•^^–^ (0–65 μM) in PBS buffer solutions (10 mM, pH = 7.4) after 5 min. (C) Fluorescence intensity at 470 nm of UDP (25 μM) as a function of O_2_^•^^–^ level. (D) Selectivity of UDP to common interfering substances (1–19: Blank, 1 mM Cys, 1 mM GSH, 1 mM Hcy, 10 mM K^+^, 10 mM Na^+^, 200 μM Ca^2+^, 200 μM Mg^2+^, 200 μM Cu^2+^, 200 μM Fe^3+^, 50 μM NO^•^, 100 μΜ ^•^OH, 10 mM H_2_O_2_, 100 μM ^1^O_2_, 100 μΜ TBHP, 100 μΜ ROO^•^, 100 μM NaClO, 25 μM ONOO^–^, and 65 μM O_2_^•^^–^) in the O_2_^•^^–^ channel. (E) Fluorescence spectra of UDP (25 μM) after incubation with different concentrations of ATP (0–22 mM) in PBS buffer solutions (10 mM, pH = 7.4) after 25 min. (F) Fluorescence intensity at 588 nm of UDP (25 μM) as a function of ATP level. (G) Selectivity of UDP to common interfering substances (1–19: Blank, 1 mM Cys, 1 mM GSH, 10 mM K^+^, 10 mM Na^+^, 200 μM Ca^2+^, 200 μM Mg^2+^, 200 μM Zn^2+^, 10 mM PO_4_^3–^, 10 mM HPO_4_^2–^, 10 mM H_2_PO_4_^–^, 10 mM SO_4_^2–^, 10 mM CO_3_^2–^, 10 mΜ UTP, 10 mΜ CTP, 10 mM GTP, 10 mM AMP, 10 mM ADP, and 10 mM ATP) in ATP channel. λ_ex_ = 380 nm for O_2_^•^^–^. λ_ex_ = 520 nm for ATP.

To meet these criteria, inspired by our previous work, caffeic acid and rhodamine B were selected as O_2_^•^^–^- and ATP-specific recognition groups and corresponding fluorophores, respectively, which were linked to diethylenetriamine (Figure 1A). Caffeic acid is a common O_2_^•^^–^ scavenger and can be used as a highly specific recognition moiety for O_2_^•^^–^, where O_2_^•^^–^ specifically oxidizes pyrocatechol to produce benzoquinone, thus promoting the production of blue fluorescence.^33,34^ Rhodamine spirolactam derivatives modified with a diethylenetriamine binding site have been shown to be highly sensitive and selective for ATP due to its multiple amino groups.^25,29^ The phosphate groups in ATP bind to several amino groups to form hydrogen bonds, and the π–π interaction between adenine in ATP and the xanthene in rhodamine synergistically triggers the opening of the nonfluorescent rhodamine spirolactam ring, which leads to the enhancement of red fluorescence.^26,28^ Meanwhile, both the reaction of the caffeic acid group with O_2_^•^^–^ and the binding response of rhodamine spirolactam with ATP are reversible, which should enable the dynamic tracking of fluctuations of O_2_^•^^–^ and ATP.^12,27^ Furthermore, the blue fluorescence channel from caffeic acid (λ_ex_ = 380 nm, λ_em_ = 470 nm) selectively activated by O_2_^•^^–^ is well separated from the red fluorescence channel of rhodamine (λ_ex_ = 520 nm, λ_em_ = 588 nm), which is specifically opened by ATP, and the maximum fluorescence emission peak difference is ∼118 nm. As such, spectral crosstalk was significantly avoided. UDP was successfully synthesized (Scheme S1) and comprehensively characterized by high-resolution mass spectrometry, ^1^H NMR, and ^13^C NMR (Figures S18–S20). The purity of UDP was calculated to be 98% as determined by HPLC analysis (Figure S21).^35,36^

### Optical Properties of UDP toward O_2_•^–^ and ATP

**Figure 2:**
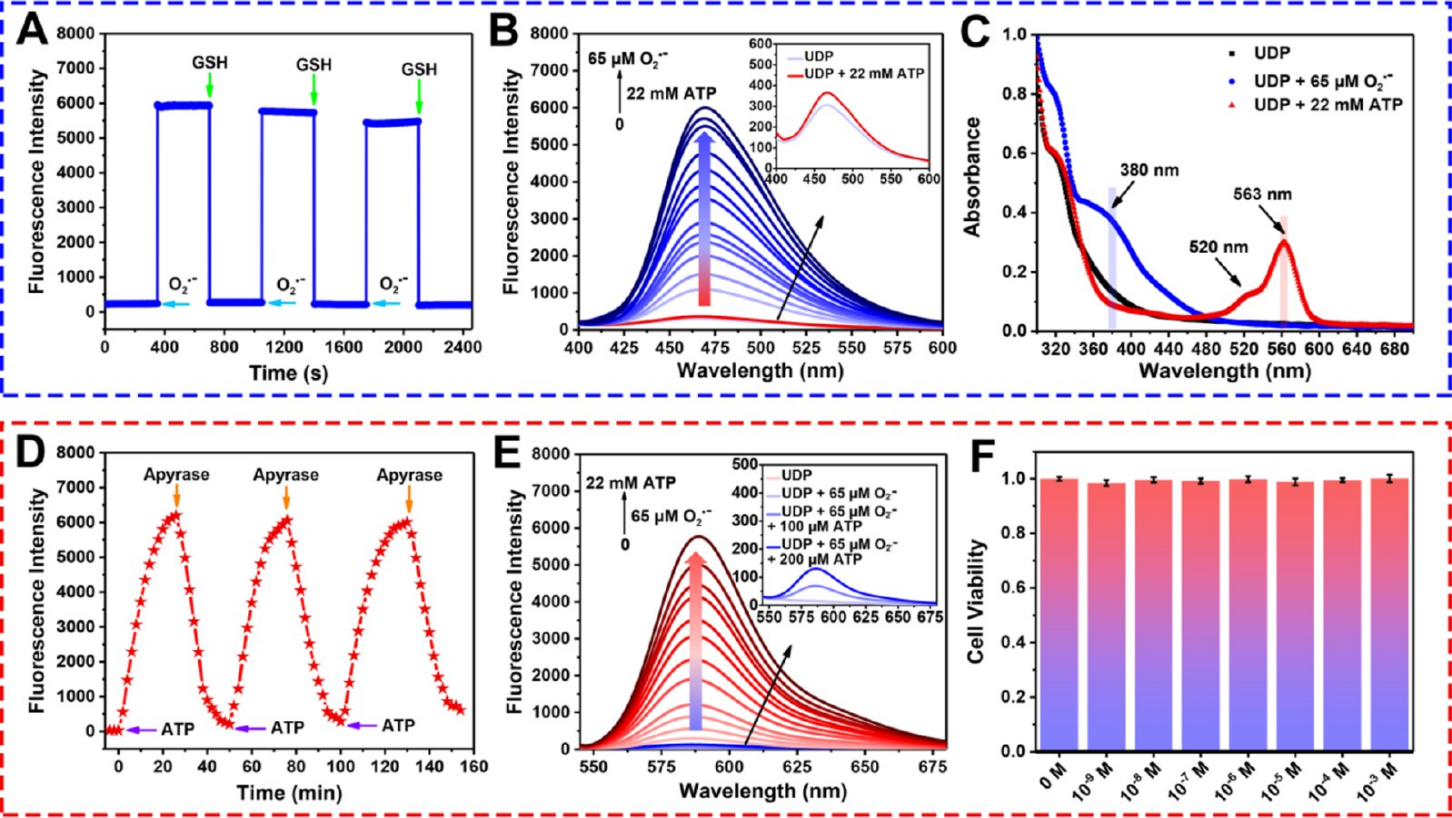
Reversibility, spectral crosstalk and biotoxicity evaluation of UDP. (A) Reversible response cycle for blue fluorescence of UDP (25 μM) with the addition of O_2_^•^^–^ (65 μM) and GSH (200 μM). λ_ex/em_ = 380/470 nm. (B) In the presence of 22 mM ATP, fluorescence spectra of UDP (25 μM) after addition of different concentrations of O_2_^•^^–^ (0–65 μM) in the O_2_^•^^–^ channel. Inset: fluorescence spectra of UDP before and after adding 22 mM ATP in the O_2_^•^^–^ channel. λ_ex_ = 380 nm. (C) Absorption spectra of UDP (25 μM) before and after reaction with 65 μM O_2_^•^^–^ and 22 mM ATP. (D) Reversible response cycle for red fluorescence of UDP (25 μM) with the addition of ATP (22 mM) and apyrase (1 U/N). λ_ex/em_ = 520/588 nm. (E) In the presence of 65 μM O_2_^•^^–^, fluorescence spectra of UDP (25 μM) after addition of different concentrations of ATP (0–22 mM) in the ATP channel. Inset: fluorescence spectra of UDP before and after adding of 65 μM O_2_^•^^–^ and the subsequent addition of 100 μM, 200 μM ATP in ATP channel. λ_ex_ = 520 nm. (F) Cell viability of HL-7702 cells after incubation with different concentrations of UDP (0–10^–3^ M).

In order to demonstrate that UDP is a two-color probe in response to O_2_^•^^–^ and ATP, the optical properties of UDP were investigated under simulated physiological conditions. First, the absorption spectrum and concentration-dependent fluorescence spectrum of UDP on the addition of various concentrations of O_2_^•^^–^ were measured in PBS buffer solutions. As shown in Figure 2C, the absorption peak at 380 nm was significantly increased upon reaction with O_2_^•^^–^, indicating that pyrocatechol was converted to benzoquinone by O_2_^•^^–^ oxidation. Under excitation at 380 nm, the blue fluorescence intensity at 470 nm gradually enhanced with an increase of O_2_^•^^–^ concentrations (0–65 μM), reaching a plateau when the O_2_^•^^–^ concentrations were further increased from 65 to 80 μM (Figure 1B). Notably, 65 μM O_2_^•^^–^ resulted in a 26-fold increase in the blue fluorescence of UDP at 470 nm (Figure S1). The blue fluorescence intensity at 470 nm exhibited a good linear relationship with concentrations of O_2_^•^^–^ (0–65 μM), and the linear equation was *F*_470nm_ = 89.99[O_2_^•^^–^](μM) + 318.72 with a linear correlation coefficient of 0.997 (Figure 1C). The limit of detection (LOD) for O_2_^•^^–^ was calculated to be as low as 34 nM (3σ/*K*, σ is the standard deviation of the blank sample and *K* is the slope of the calibration curve), suggesting highly sensitive detection of O_2_^•^^–^ by UDP in vitro. We then evaluated the anti-interference performance of UDP toward O_2_^•^^–^. Only O_2_^•^^–^ could trigger a significant enhancement of blue fluorescence without interference from other abundant ROS, RNS, reducing substances, and metal ions found in organisms (Figure 1D). We then examined the blue fluorescence response of UDP with the coexistence of O_2_^•^^–^ and other various ROS, RNS, and metal ions (Figure S2). In vitro experiments indicated that blue fluorescence was enhanced after mixing with O_2_^•^^–^, further confirming that UDP could be utilized as a potential tool for the specific detection of O_2_^•^^–^ in complex biological systems.

The reactivity of UDP with ATP was then evaluated. When ATP was added, a maximum absorption peak at 563 nm accompanied by a flat absorption peak at 520 nm was observed due to the ATP-induced ring opening of the spirolactam of rhodamine (Figure 2C). In the presence of various concentrations of ATP (0–22 mM), the red fluorescence gradually enhanced and peaked at 588 nm (Figure 1E). Additionally, UDP exhibited a 204-fold enhancement of red fluorescence at 588 nm after incubation with 22 mM ATP (Figure S3). The fluorescence intensity centered at 588 nm exhibited a linear increase with ATP concentrations in the range of 0–22 mM. The linear correlation equation was *F*_588nm_ = 256.51[ATP](mM) + 41.13 with a linear correlation coefficient of 0.997 (Figure 1F). The LOD for ATP was calculated to be 14 μM. Furthermore, UDP exhibited higher selectivity for ATP over other biological species, including adenosine phosphates, nucleoside triphosphates, amino acids, and anions/cations (Figures 1G, S4). As such, the above in vitro experiments confirmed that UDP had significant potential for ATP biosensing.

We then investigated the reversible responses of UDP to O_2_^•^^–^ and ATP and spectral crosstalk. Figure 2A indicates that the blue fluorescence activated by O_2_^•^^–^ could be effectively quenched by the reducing substance glutathione (GSH), and the subsequent addition of O_2_^•^^–^ could restore the blue fluorescence. The reversible response of UDP with O_2_^•^^–^ was stable for three repeated cycles. Similarly, the red fluorescence of UDP induced by ATP could also be significantly suppressed by apyrase (ATP hydrolase), further demonstrating the ability of UDP to monitor the dynamic changes of O_2_^•^^–^ and ATP in vitro (Figure 2D). Subsequently, the fluorescence response of UDP to ATP in the O_2_^•^^–^ channel was examined. As shown in Figure 2B, when 22 mM ATP was added, blue fluorescence did not change significantly, indicating that the O_2_^•^^–^ channel could not collect the fluorescence signal of UDP in response to ATP. In the presence of 22 mM ATP, the subsequent addition of different concentrations of O_2_^•^^–^ triggered significantly enhanced blue fluorescence signals in the O_2_^•^^–^ channel, demonstrating that the O_2_^•^^–^ channel could independently and sensitively capture the changes of O_2_^•^^–^. We then determined whether the response of UDP to O_2_^•^^–^ could be observed in the ATP channel. It was worth noting that the addition of 65 μM O_2_^•^^–^ failed to cause a significant enhancement of red fluorescence (Figure 2E). However, in the presence of 65 μM O_2_^•^^–^, the subsequent addition of different concentrations of ATP led to the gradual increase of red fluorescence, suggesting that the ATP channel does not respond to changes of O_2_^•^^–^ and could only collect signals associated with ATP. The above results confirmed that O_2_^•^^–^ and ATP could be independently monitored using their respective channels without spectral crosstalk. Figure S5 confirms pH insensitivity of UDP in response to O_2_^•^^–^ over the physiological pH range, and 7.4 was the optimal pH value of UDP in response to ATP (Figure S6). The kinetics of UDP in response to O_2_^•^^–^ and ATP were examined to confirm the efficacy of UDP. As a result, when 65 μM O_2_^•^^–^ was added, UDP immediately exhibited an enhanced emission intensity at 470 nm and rapidly achieved equilibrium (Figure S7). With the addition of 10 mM ATP, the fluorescence intensity at 588 nm increased gradually and the intensity reached a stable maximum within 23 min (Figure S8). After reacting with O_2_^•^^–^ or ATP, the fluorescence intensities at 470 and 588 nm were almost unchanged, which indicated that UDP exhibits good photostability. Taken together, these results confirm that UDP exhibits high sensitivity, excellent selectivity, reversibility, and good photostability as well as resistance to pH interference without spectral crosstalk in response to O_2_^•^^–^ and ATP, making UDP suitable for the simultaneous detection of O_2_^•^^–^ and ATP in vivo during HIRI.

### Simultaneous Imaging and Dynamic Monitoring of Endogenous O_2_•^–^ and ATP Fluctuations in Hepatocytes

Having confirmed the excellent performance of UDP in response to O_2_^•^^–^ and ATP in vitro, the feasibility of using UDP for the simultaneous imaging and dynamic monitoring of endogenous O_2_^•^^–^ and ATP was investigated in hepatocytes. First, the potential biological toxicity of UDP was comprehensively evaluated in hepatocytes. Figure 2F confirms that the viability of hepatocytes reached more than 95% after 24 h incubation of UDP, illustrating low toxicity of UDP which was suitable for cell imaging. To represent O_2_^•^^–^ and ATP signaling via two distinguishable blue and red channels, spectral crosstalk was first examined between the O_2_^•^^–^ and ATP channels. Blue fluorescence channel (λ_ex_ = 405 nm, λ_em_ = 420–490 nm) for O_2_^•^^–^ and red fluorescence channel (λ_ex_ = 514 nm, λ_em_ = 525–668 nm) for ATP were selected, plus channel 3 (λ_ex_ = 405 nm, λ_em_ = 525–668 nm) to exclude spectral crosstalk. As shown in Figure S9, the blue fluorescence and red fluorescence in normal hepatocytes represented endogenous O_2_^•^^–^ and ATP levels, respectively, whereas channel 3 showed negligible green fluorescence. 2-ME promoted O_2_^•^^–^ production by inhibiting the activity of superoxide dismutase (SOD).^37^ After incubation with 2-ME and ATP in hepatocytes, both blue fluorescence and red fluorescence were enhanced, while the green fluorescence in channel 3 was negligible, which implied that there was no spectral crosstalk between the blue and red channels or interference from fluorescence resonance energy transfer (FRET).

**Figure 3:**
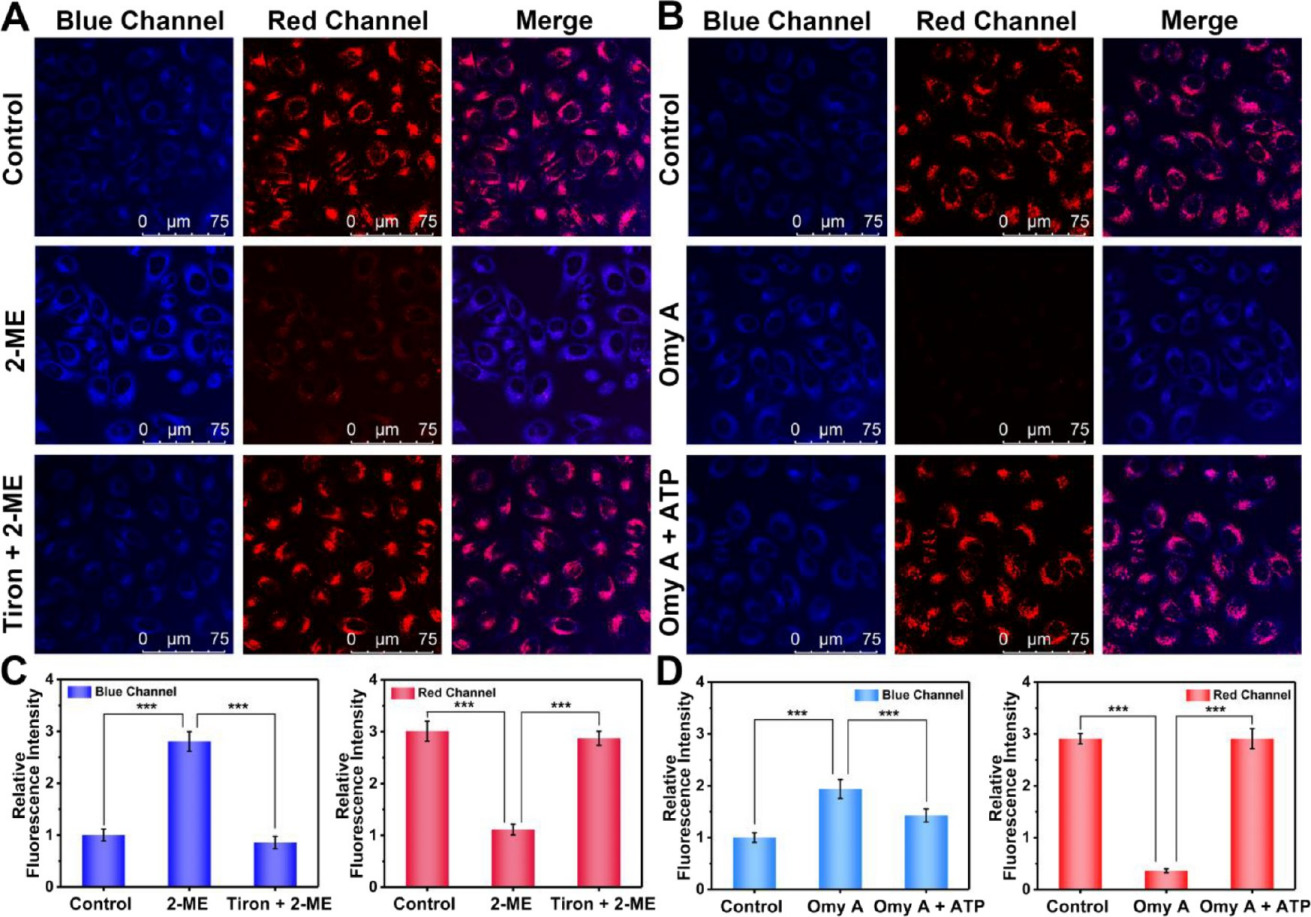
Confocal fluorescence imaging of O_2_^•^^–^ and ATP fluctuations in hepatocytes stimulated by 2-ME or oligomycin A. (A) Confocal fluorescence images of O_2_^•^^–^ (blue channel, λ_ex_ = 405 nm, λ_em_ = 420–490 nm) and ATP (red channel, λ_ex_ = 514 nm, λ_em_ = 525–668 nm) in hepatocytes by UDP staining (40 μM, 20 min) after incubation of 2-ME (3 μg/mL, 1 h) and Tiron (10 μM, 1 h). (B) Confocal fluorescence images of O_2_^•^^–^ (blue channel, λ_ex_ = 405 nm, λ_em_ = 420–490 nm) and ATP (red channel, λ_ex_ = 514 nm, λ_em_ = 525–668 nm) in hepatocytes by UDP staining (40 μM, 20 min) after incubation of oligomycin A (50 μM, 1 h) and ATP (10 mM, 1 h). (C, D) Relative blue and red fluorescence intensity output of (A) and (B), respectively. The blue fluorescence intensity of control group was defined as 1. The data are expressed as the mean ± SD. ***P < 0.001. Concordant results were obtained from five independent experiments.

We then turned our attention to the imaging of endogenous O_2_^•^^–^ and ATP levels in hepatocytes stimulated by exogenous substances using confocal fluorescence microscopy. As shown in Figure 3A, after incubation with 2-ME for 1 h, hepatocytes exhibited a 2.8-fold O_2_^•^^–^-associated blue fluorescence enhancement and a 0.37-fold ATP-related red fluorescence reduction in comparison to the control group. However, after pretreatment with Tiron (O_2_^•^^–^ scavenger) and subsequent incubation with 2-ME,^38^ the blue fluorescence was reduced and the red fluorescence was conversely elevated (Figure 3C). We noted that the accumulation of endogenous O_2_^•^^–^ in hepatocytes could reduce intracellular ATP levels, implying that intracellular O_2_^•^^–^ excess could regulate ATP fluxes. We also evaluated whether an intracellular imbalance of energy metabolism could affect redox homeostasis (Figure 3B). Oligomycin A, as an inhibitor of F_o_F_1_-ATPase, could block ATP synthesis.^39^ After treatment with oligomycin A for 1 h, a 0.12-fold reduction of red fluorescence and a 1.9-fold elevation of blue fluorescence were observed in hepatocytes (Figure 3D). However, when exogenous ATP was added, the red fluorescence of hepatocytes was restored, while the blue fluorescence was decreased. These results are consistent with previous reports that the failure of ATP synthesis caused by oligomycin A could lead to intracellular redox imbalance, thus resulting in the increase of O_2_^•^^–^ to a certain extent, and the supplement of appropriate amount of ATP could recover the energy metabolism and redox imbalance.^25,26^ In addition, the ability of UDP to respond reversibly to O_2_^•^^–^ and ATP was also verified in hepatocytes (Figure S10). Hence, these results unequivocally demonstrated that UDP could function as a powerful tool for the real-time visualization and reversible monitoring of endogenous O_2_^•^^–^ and ATP variations in hepatocytes. The intimate correlation between oxidative stress and energy metabolism in hepatocytes was confirmed via the differentiated blue fluorescence signal for O_2_^•^^–^ and red fluorescence signal for ATP.

### Real-Time Visualization of O_2_•^–^ and ATP Dynamics in Hepatocytes in the Process of HIRI and the Effect of HIRI Drug Intervention

We next turned our attention to the dynamic imaging of O_2_^•^^–^ and ATP fluxes in living HIRI cells. Existing evidence suggests that the pathological mechanism of HIRI is intimately linked with oxidative stress and energy metabolism.^40,41^ Endogenous O_2_^•^^–^ and ATP can be applied as promising, in situ hallmarks that are closely related to oxidative stress and energy conversion, respectively.^4,13^ Hence, we attempted to simultaneously detect HIRI with the aid of UDP in response to O_2_^•^^–^ and ATP.

**Figure 4:**
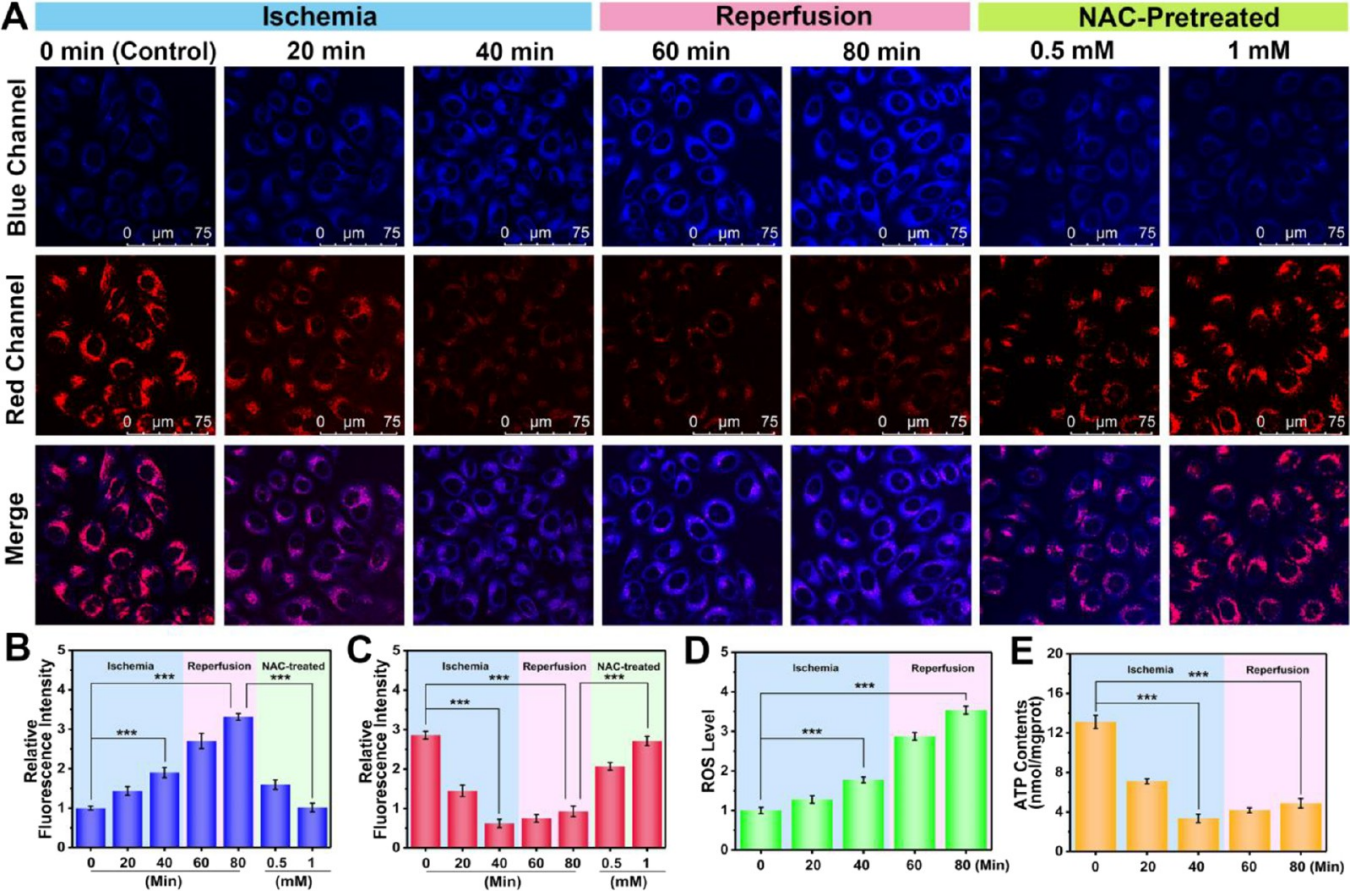
Dynamic visualization of O_2_^•^^–^ and ATP fluctuations in hepatocytes during the whole process of HIRI and effect of intervention by HIRI drug NAC. (A) Fluorescence imaging of O_2_^•^^–^ (blue channel, λ_ex_ = 405 nm, λ_em_ = 420–490 nm) and ATP (red channel, λ_ex_ = 514 nm, λ_em_ = 525–668 nm) by UDP (40 μM) in hepatocytes undergoing 0, 20, or 40 min of ischemia and 20 or 40 min of reperfusion after 40 min of ischemia, and pretreatment with 0.5 or 1 mM NAC. (B, C) Relative blue and red fluorescence intensity output of (A). The blue fluorescence intensity of control group was defined as 1. (D) Relative ROS levels in different phases of HIRI by ROS content assay kit. (E) ATP levels in different phases of HIRI by the ATP content assay kit. The data are expressed as the mean ± SD. ***P < 0.001. Concordant results were obtained from five independent experiments.

First, a HIRI model in hepatocytes was established by glucose-serum-oxygen deprivation and subsequent reperfusion,^42^ which was validated by a series of hepatocyte injury markers such as ALT, AST, tumor necrosis factor-α (TNF-α), and lactate dehydrogenase (LDH).^43,44^ As depicted in Figure S11, the levels of ALT and AST were significantly elevated in HIRI hepatocytes in comparison with control hepatocytes. Interestingly, increases of TNF-α and LDH release were observed in the HIRI group rather than in control group. Collectively, these results revealed significant injury in HIRI hepatocytes. In order to trace O_2_^•^^–^ and ATP in the whole process of HIRI in detail, hepatocytes were divided into five groups to monitor O_2_^•^^–^ and ATP levels in the respective phases of ischemia and reperfusion, including control group, 20 min of ischemia group, 40 min of ischemia group, 40 min of ischemia followed by 20 min of reperfusion group, and 40 min of ischemia followed by 40 min of reperfusion group. The five groups were stained with UDP, and the O_2_^•^^–^-related blue fluorescence signal and ATP-representative red fluorescence signal were captured. As illustrated in Figure 4A, with the prolongation of ischemia, the red fluorescence of hepatocytes decreased, while the blue fluorescence showed a slight upward trend. A 0.22-fold decrease of red fluorescence and a 1.9-fold enhancement of blue fluorescence were observed in the 40 min of ischemia group compared to normal cells, indicating that a dramatic decrease of ATP and an increase of O_2_^•^^–^ (Figure 4B,C). In the case of ischemia, ATP could only be produced through anaerobic glycolysis due to the inhibition of oxidative phosphorylation, leading to the depletion of ATP.^45^ Intracellular hypoxia provoked the production of ROS through complex III of the mitochondrial electron transport chain during the ischemia phase.^46,47^ After hepatocytes undergoing ischemia were subjected to reperfusion, the blue fluorescence increased significantly (Figure 4A). The blue fluorescence was enhanced 3.3-fold in the hepatocytes subjected to 40 min of ischemia and 40 min of reperfusion in comparison to the control group (Figure 4B). This phenomenon was attributed to a burst of O_2_^•^^–^ during reperfusion.^48^ Compared with hepatocytes subjected to ischemia for 40 min, the subsequent reperfusion resulted in a slight recovery of red fluorescence, but the ATP level was still significantly lower than that of the normal group (Figure 4A). The red fluorescence of hepatocytes undergoing 40 min of ischemia and 40 min of reperfusion was 0.32 times lower than that of normal hepatocytes (Figure 4C). The slight increase of the ATP level during reperfusion was related to the recovery of oxidative phosphorylation in hepatocytes.^45^ We speculate that the O_2_^•^^–^ outburst and the leakage of adenine nucleotides affect the recovery of the ATP levels, resulting in the slight recovery of ATP during reperfusion.^49^ In order to image in situ O_2_^•^^–^ and ATP dynamics for the process of HIRI, the blue fluorescence for O_2_^•^^–^ signaling and red fluorescence for ATP signaling of hepatocytes in the same region were captured in real-time (Figure S12).

To verify the simultaneous imaging capability of UDP for O_2_^•^^–^ and ATP in HIRI hepatocytes and the reliability of the variations of O_2_^•^^–^ and ATP during HIRI, commercial ROS and ATP assay kits were used to evaluate ROS and ATP levels in the whole process of HIRI. 2′,7′-dichlorodihydrofluorescein diacetate (DCFH-DA) could be oxidized by ROS to produce fluorescent 2′,7′-dichlorofluorescein (DCF), which was used to image ROS in HIRI.^50^ We found that ROS tended to rise gradually during ischemia, while a large explosion of ROS was observed during reperfusion (Figures 4D, S13). ATP concentrations decreased sharply from 13 to 3.3 nmol/mgprot in cells after 40 min of ischemia, whereas ATP levels increased slowly from 3.3 to 4.9 nmol/mgprot in hepatocytes that received reperfusion for 40 min (Figure 4E). The determination of ROS and ATP levels measured by combining commercial ROS and ATP kits during HIRI was consistent with a fluorescence imaging phenomenon using UDP. Compared with commercial ROS and ATP kits for the determination of ROS and ATP contents in HIRI separately, UDP integrated both functions and provided effective and real-time information for HIRI conveniently by simultaneously detecting O_2_^•^^–^ and ATP. Therefore, these results confirmed the imaging capacity of UDP for the detection of O_2_^•^^–^ and ATP in hepatocytes during HIRI.

Having demonstrated the system works for the detection of HIRI, we then evaluated the intervention of HIRI drugs using the synergistic changes of blue and red fluorescence. *N*-acetylcysteine (NAC) has been reported as an effective drug commonly used to protect the liver from HIRI by directly removing ROS.^51^ For HIRI hepatocytes pretreated with 0.5 mM NAC for 1 h, the blue fluorescence was clearly reduced, while the red fluorescence was significantly restored (Figure 4A). Additionally, the blue fluorescence of HIRI hepatocytes pretreated with 1 mM NAC was reduced by 0.31 times and the red fluorescence was enhanced by 2.9 times compared with HIRI hepatocytes without NAC intervention (Figure 4B,C), which indicated that NAC intervention could remove O_2_^•^^–^ and restore the levels of ATP during HIRI. Hence, the above results confirmed that UDP can provide dynamic visualization of O_2_^•^^–^ and ATP fluctuations in hepatocytes during HIRI and could provide effective evaluation of the effect of NAC.

### Real-Time Imaging of O_2_•^–^ and ATP in Mouse Livers during HIRI

**Figure 5:**
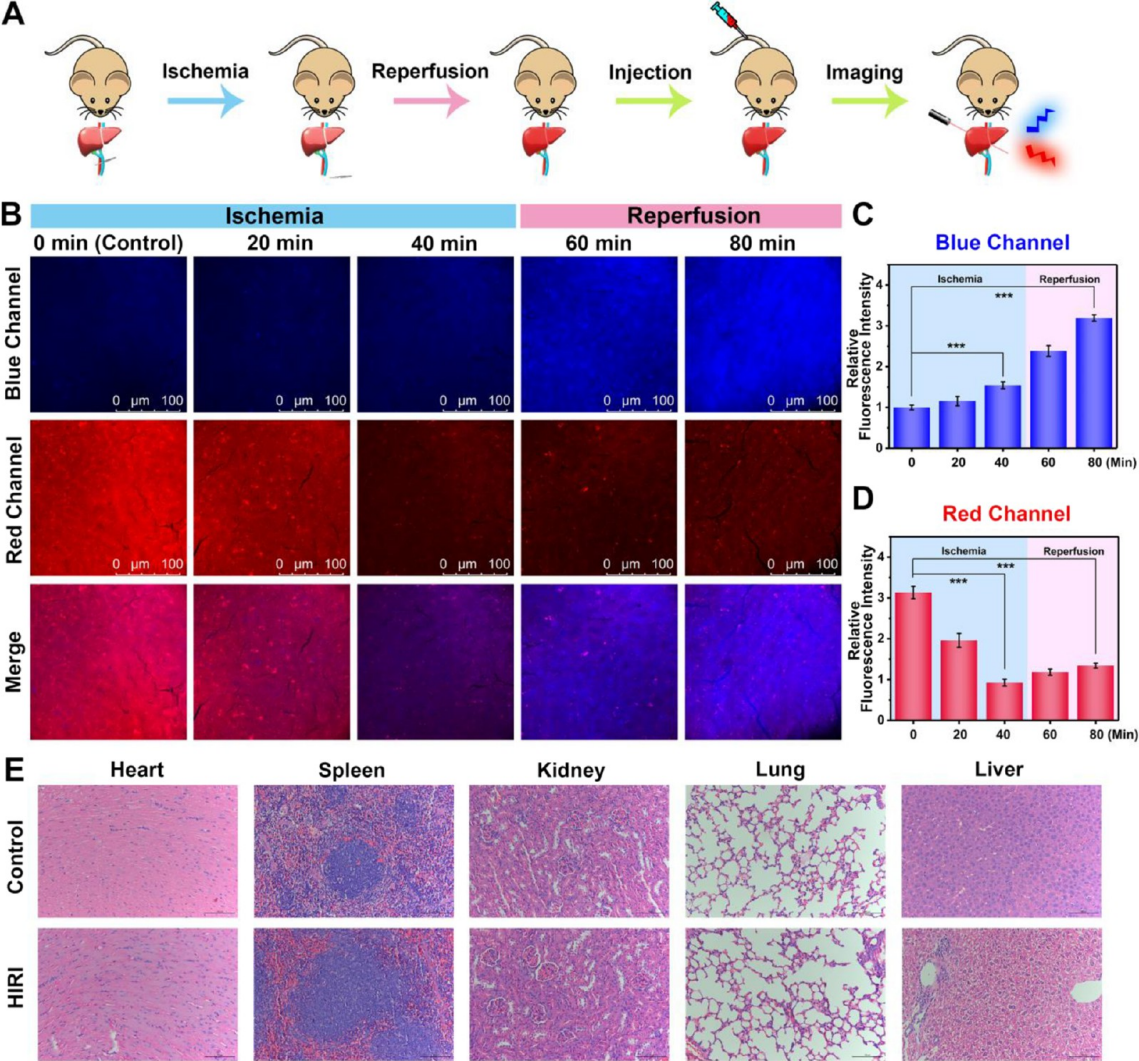
Real-time visualization of O_2_^•^^–^ and ATP dynamics in mouse livers during HIRI. (A) Procedure diagram. (B) Fluorescence imaging of O_2_^•^^–^ (blue channel, λ_ex_ = 405 nm, λ_em_ = 420–490 nm) and ATP (red channel, λ_ex_ = 514 nm, λ_em_ = 525–668 nm) by UDP (100 μM) in mouse livers undergoing 0, 20, or 40 min of ischemia and 20 or 40 min of reperfusion after 40 min of ischemia. (C, D) Relative blue and red fluorescence intensity output of (B). The blue fluorescence intensity of the control group was defined as 1. (E) H&E staining of heart, spleen, kidney, lung, and liver in control and HIRI mice. The data are expressed as the mean ± SD. ***P < 0.001. Concordant results were obtained from five independent experiments.

First, the real-time visualization of O_2_^•^^–^ and ATP dynamics were examined in the livers of mice. As shown in Figure S14, the blue fluorescence was increased after the intraperitoneal injection of 2-ME and subsequent injection of GSH resulted in a decrease in O_2_^•^^–^-associated blue fluorescence, which demonstrated the reversible detection of UDP toward O_2_^•^^–^. Similarly, it was discovered that the red fluorescence remarkably enhanced upon the injection of ATP, and successive injection of apyrase resulted in a restoration of red fluorescence (Figure S15). These results indicated that UDP could serve as a robust tool for reversible detection of the changes in O_2_^•^^–^ and ATP in the livers of mice. Afterward, the performance of UDP-mediated fluorescence visualization of O_2_^•^^–^ and ATP in mouse livers throughout the HIRI process was systematically investigated in a HIRI mouse model. HIRI models were established in mice according to the previously reported method, in which microvessel clamps were used to block the hepatic artery and portal vein blood supply, resulting in approximately 70% blood flow deprivation in the liver, followed by the removal of the microvessel clamps for the reperfusion process.^50^ The determination of serum ALT, AST, and TNF-α levels were investigated. It was discovered that ALT, AST, and TNF-α levels were significantly increased in the serum of mice subjected to HIRI, indicating the HIRI mice suffered severe hepatic injury (Figure S11). In order to image O_2_^•^^–^ and ATP fluctuations for the whole process of HIRI, mice were divided into five groups consisting of control group, 20 min of ischemia group, 40 min of ischemia group, 40 min of ischemia followed by 20 min of reperfusion group, and 40 min of ischemia followed by 40 min of reperfusion group. The five groups of mice were then injected with UDP through the tail vein, and the blue and red fluorescence signals in the livers were collected (Figure 5A). As shown in Figure 5B, with the extension of ischemia, the red fluorescence exhibits a significant decline, while the blue fluorescence underwent a minor increase, suggesting that the ATP content in the livers decreased sharply during ischemia and the O_2_^•^^–^ levels did not increase dramatically. Compared with the normal group, a 0.30-times decrease of red fluorescence and 1.5-times increase of blue fluorescence were observed in the 40 min of ischemia group (Figure 5C,D). The subsequent reperfusion resulted in a conspicuously enhanced blue fluorescence for O_2_^•^^–^ and a slightly elevated red fluorescence for ATP in comparison to mice subjected to 40 min of ischemia, suggesting that a rapid accumulation of O_2_^•^^–^ occurred during reperfusion while ATP was slightly upregulated. The blue fluorescence and red fluorescence in livers of mice for 40 min of ischemia followed by 40 min of reperfusion were 3.2-fold and 0.43-fold that of the control group, respectively (Figure 5C,D). As far as we know, this research represents the first simultaneous and real-time imaging of O_2_^•^^–^ and ATP dynamics in HIRI mouse livers. Body weight tests of mice after intraperitoneal injection with UDP and hematoxylin and eosin (H&E) staining of the major organs were performed to evaluate the biocompatibility of UDP. The UDP-treated group of mice showed no apparent difference with the trend of body weight for the control group which received saline (Figure S16). After injection with UDP, no obvious damage was observed in the heart, liver, spleen, kidney, and lung tissues, indicating UDP exhibited good biocompatibility (Figure S17). Furthermore, after intraperitoneal injection with UDP, H&E staining of major organ tissues in the control group and HIRI groups of mice was conducted to identify the histological changes. As a result, there was no evident injury in the heart, liver, spleen, kidney, and lung tissues of the control group, whereas in the liver tissues of HIRI mice, the cytoplasm was lightly stained, and there were a few lymphocytes and granulocytes clustered near the portal area, demonstrating liver injury occurred in HIRI mice (Figure 5E). Therefore, these results confirmed that UDP was suitable as a robust in vivo bioimaging tool for the real-time and dynamic visualization of O_2_^•^^–^ and ATP in HIRI mouse livers.

### Potential Signaling Pathways Mediating O_2_•^–^ and ATP Changes in the HIRI Process

In the above experiments, we found that the synergistic variation of O_2_^•^^–^ and ATP could be successfully visualized during HIRI with the aid of UDP. Subsequently, the potential signaling pathways mediated by O_2_^•^^–^ and ATP changes during HIRI were comprehensively investigated. It has been reported that ATP decomposes into hypoxanthine during ischemia.^52^ During reperfusion, hypoxanthine undergoes enzymatic reaction with O_2_ in tissues in the presence of xanthine oxidase, resulting in an outburst of O_2_^•^^–^, indicating that a depletion of ATP in HIRI can lead to the production of O_2_^•^^–^.^49,53−55^ However, it is not clear whether O_2_^•^^–^ can regulate ATP fluctuations in HIRI. The above experiments showed that after the intervention of NAC in HIRI hepatocytes, ATP fluxes also changed significantly in addition to the obvious changes in O_2_^•^^–^ levels, which indicated that O_2_^•^^–^ could affect the ATP level in HIRI as well. Together with the evidence that significantly reduced ATP red fluorescence was detected in 2-ME-stimulated O_2_^•^^–^ producing hepatocytes, it was further suggested that endogenous accumulation of O_2_^•^^–^ could reduce the intracellular ATP level. Therefore, we then specifically explored the signaling pathway of O_2_^•^^–^ influencing ATP-level changes during HIRI.

### Excess O_2_•^–^ Inactivated Succinate Dehydrogenase and Thus Affected Intracellular ATP Levels in HIRI

Under physiological conditions, ATP in the liver is mainly generated through oxidative phosphorylation in the electron transport chain of mitochondria, which jointly coordinates with the tricarboxylic acid (TCA) cycle to constitute the bioproductive activities of mitochondria.^56^ The TCA cycle extracts energy from substrates to generate NADH, which is then oxidized in the electron transport chain, promoting oxidative phosphorylation to produce ATP.^57^ SDH not only acts as a pivotal enzyme in the TCA cycle but also serves as mitochondrial complex II in the oxidative phosphorylation, catalyzing the oxidation of succinate to fumarate and the reduction of ubiquinone (UQ) to ubiquinol (UQH_2_).^58^ As a central hub connecting oxidative phosphorylation with the TCA cycle, SDH is indispensable in regulating intracellular ATP levels.^59,60^ Thus, we speculated that excess O_2_^•^^–^ in HIRI may regulate intracellular ATP levels by affecting the activity of SDH. An SDH activity kit was used to assess SDH activity of HIRI hepatocytes and normal hepatocytes. As illustrated in Figure 7A, the SDH activity of HIRI hepatocytes was significantly lower than that of normal hepatocytes, but the SDH activity was recovered after the addition of NAC to remove O_2_^•^^–^. In order to prove that O_2_^•^^–^ is responsible for inactivation of SDH, we used 2-ME to stimulate the production of O_2_^•^^–^, and found that the activity of SDH was downregulated, while the activity of SDH was distinctly enhanced in hepatocytes pretreated with Tiron. These results confirmed that excessive O_2_^•^^–^ inactivated SDH in HIRI hepatocytes.

Next, we investigated the effect of SDH activity on intracellular ATP levels. Normal cells and HIRI cells were incubated with 3-nitropropionic acid (3-NPA), which is an inhibitor of SDH (Figure 7E).^59,60^ Intracellular ATP levels were evaluated using UDP. The results indicated that the ATP-related red fluorescence was significantly reduced in the normal cells treated with 3-NPA compared with normal cells, indicating that the inactivation of SDH could lead to a depletion of intracellular ATP. Furthermore, it was observed that the ATP level in HIRI cells was low, and the 3-NPA treated HIRI cells showed a lower ATP level (Figure 7F). As such data from the SDH activity kit and fluorescence imaging data from UDP indicated that excessive O_2_^•^^–^ inactivated SDH in HIRI, thereby affecting intracellular ATP fluxes.

### O_2_•^–^ Oxidized Histidine, Cysteine, Methionine, and Tryptophan Residues in SDH

**Figure 6:**
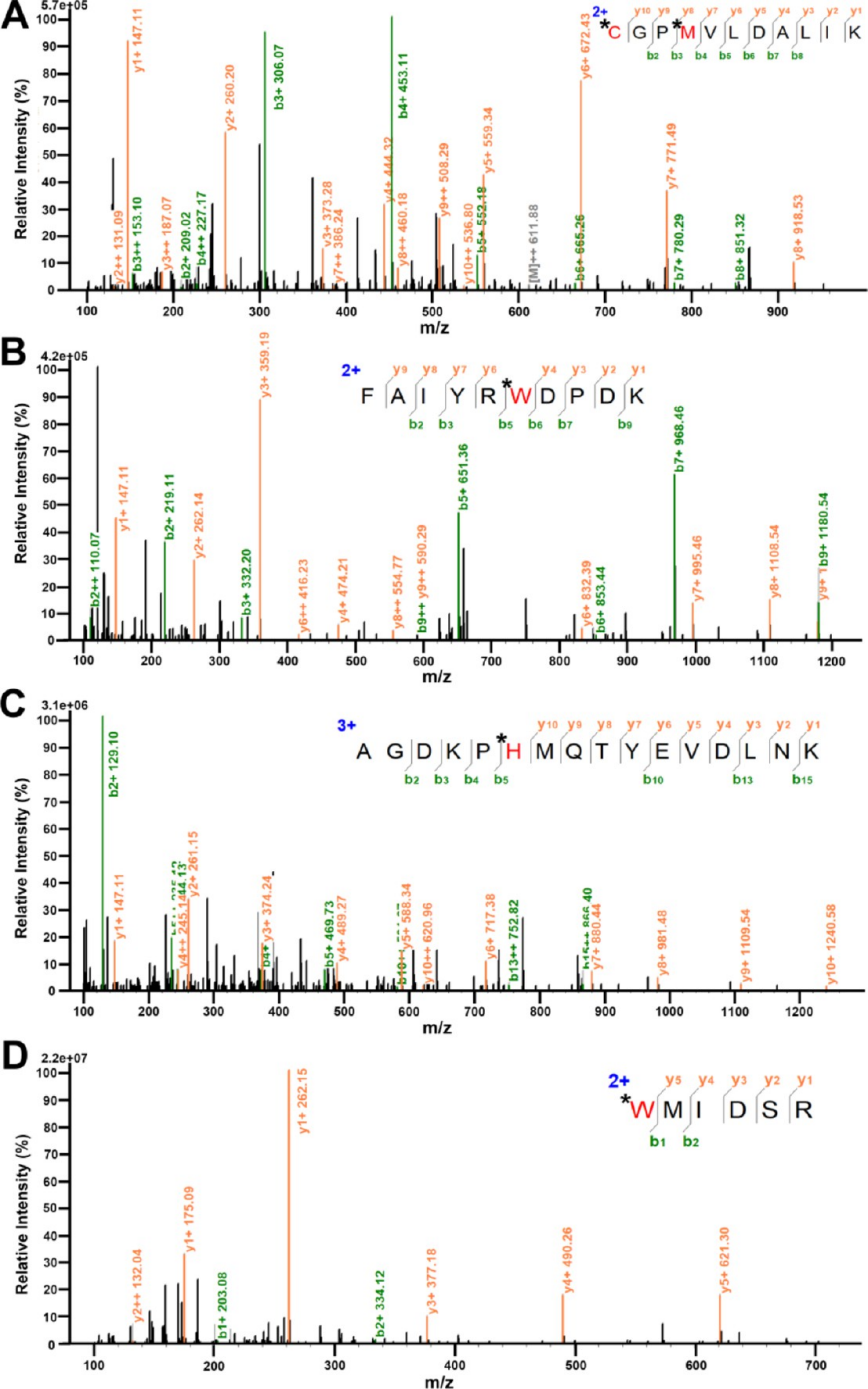
Proteomic analysis of the reaction of SDH with O_2_^•^^–^ through LC–MS/MS. (A) Oxidation of C68, M71. (B) Oxidation of W47. (C) Oxidation of H57. (D) Oxidation of W218. Residues represented by * and red color are modification sites by O_2_^•^^–^.

In order to investigate the specific inactivation mechanism of SDH by O_2_^•^^–^, proteomic analysis analyzed by LC–MS was conducted on SDH to explore the post-translational modification of SDH caused by O_2_^•^^–^. As shown in Figure 6, oxidation of histidine, cysteine, methionine, and tryptophan residues in the active domain of SDH by O_2_^•^^–^ were identified.^61,62^ Therefore, we speculated that excess O_2_^•^^–^ oxidized histidine, cysteine, methionine, and tryptophan residues in SDH during HIRI, resulting in the inactivation of SDH.

### Inactivation of SDH Resulted in Reduced Mitochondrial NADH Levels in Hepatocytes during HIRI

**Figure 7:**
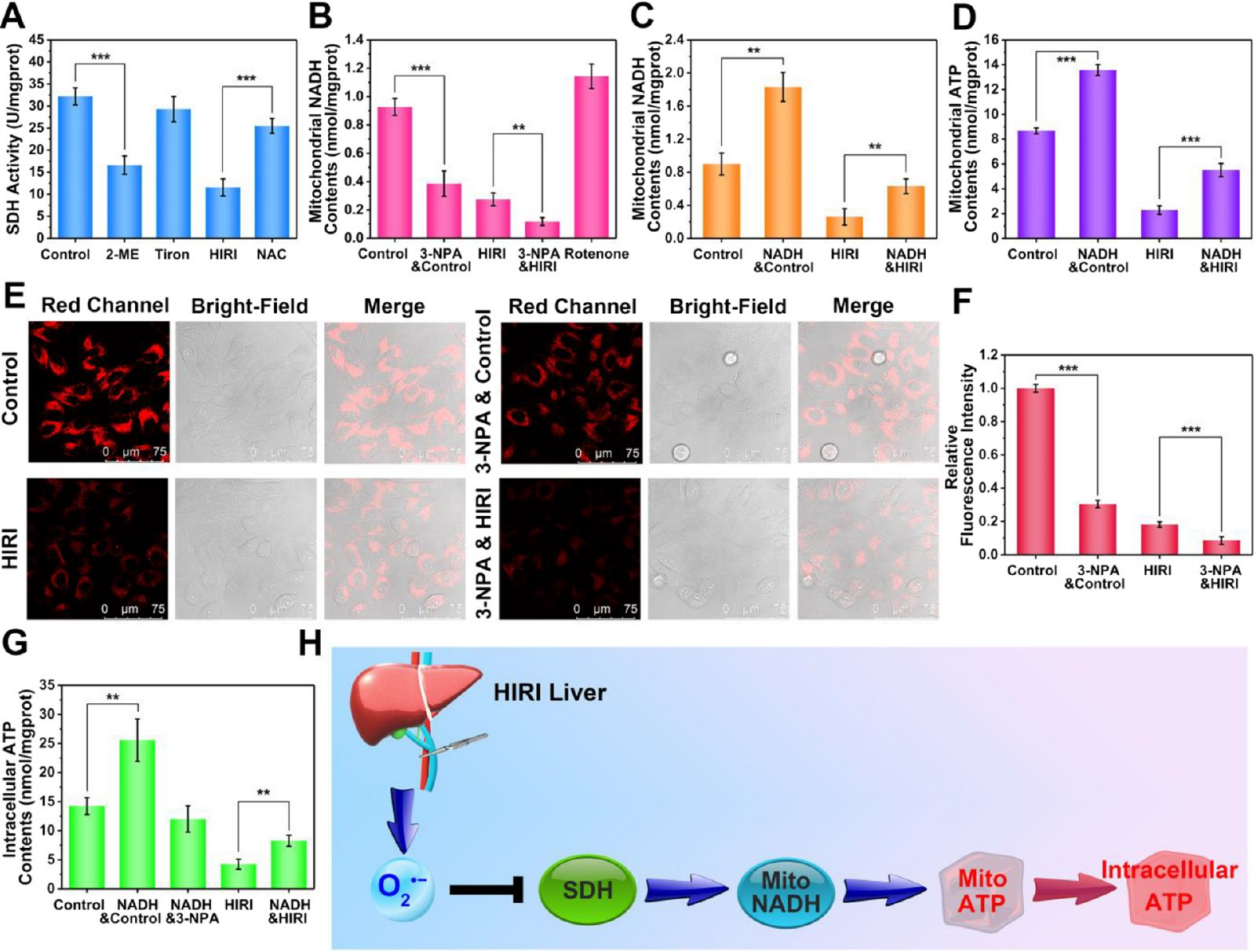
Potential signaling pathways involving O_2_^•^^–^ and ATP in HIRI. (A) SDH activity assays in hepatocytes under different treatments. (B, C) Analyses of mitochondrial NADH contents in hepatocytes under various treatments. (D) Mitochondrial ATP contents analyses of hepatocytes in different treatment groups. (E) Fluorescence imaging of ATP (red channel, λ_ex_ = 514 nm, λ_em_ = 525–668 nm) in hepatocytes by UDP under the incubation of 3-NPA. (F) Relative red fluorescence intensity output of (E). (G) Intracellular ATP contents analyses of hepatocytes in different treatment groups. (H) Schematic of intracellular O_2_^•^^–^—SDH—Mito NADH—Mito ATP—intracellular ATP cascade signaling pathway during HIRI.

Having discovered that SDH activity was significantly decreased in HIRI hepatocytes, we next focused on downstream inactivation of SDH caused by excess O_2_^•^^–^. The TCA cycle in which SDH participates is a key metabolic pathway for the production of the electron donor NADH.^63^ Electrons from NADH and succinate in the mitochondrial inner membrane pass through an electron transport chain to O_2_ and drive the synthesis of ATP.^63^ We hypothesized that SDH regulated intracellular ATP levels by influencing mitochondrial NADH contents, and then, we explored whether the activity of SDH could regulate the levels of mitochondrial NADH. Herein, we first isolated mitochondria from hepatocytes from different treatment groups using a mitochondria isolation kit and then detected NADH concentrations of the mitochondria in each group using an NADH assay kit (Figure 7B). The results indicated that the mitochondrial NADH content decreased from 0.93 to 0.39 nmol/mgprot in hepatocytes treated with the SDH inhibitor 3-NPA, indicating that the inactivation of SDH effectively downregulated mitochondrial NADH levels. Moreover, the NADH content in the mitochondria of HIRI hepatocytes was calculated to be 0.27 nmol/mgprot, implying that the inactivation of SDH led to a decrease of mitochondrial NADH levels in HIRI. Interestingly, mitochondrial NADH levels reached the lowest level in the HIRI group incubated with 3-NPA, suggesting that enhanced SDH inactivation in HIRI further reduces mitochondrial NADH concentrations. Meanwhile, rotenone, which can significantly increase mitochondrial NADH content, was selected as a positive control. These results provided direct evidence that the inactivation of SDH in HIRI resulted in the reduction of mitochondrial NADH levels in hepatocytes.

### Reduction of Mitochondrial NADH Contents Caused the Decrease of Mitochondrial and Intracellular ATP Levels during HIRI

Since NADH plays a vital role in mitochondrial bioenergetics by supplying electrons to respiratory complex I,^65^ we next investigated whether the decrease in mitochondrial NADH content in hepatocytes affected mitochondrial energy (ATP) production during HIRI. After exposure of 1 mM NADH to normal cells for 1 h, the additional NADH could increase mitochondrial NADH contents from 0.90 to 1.8 nmol/mgprot (Figure 7C), and mitochondrial ATP levels of hepatocytes rapidly increased from 8.7 to 13.6 nmol/mgprot (Figure 7D), indicating that the accumulation of NADH in the mitochondria of hepatocytes did cause the increase in mitochondrial ATP content. The concentrations of mitochondrial ATP in HIRI hepatocytes were significantly lower than that of normal cells. However, NADH treatment could effectively reverse the ATP content in the mitochondria of HIRI hepatocytes. Thus, a decrease of mitochondrial NADH content in HIRI hepatocytes could effectively downregulate mitochondrial ATP content. Glycolytic ATP synthesis could be impaired by restricted mitochondrial ATP production, in addition, obstruction of both mitochondrial and glycolytic ATP production would lead to disastrous depletion of total cellular ATP.^57^ We therefore evaluated the effect of mitochondrial NADH content on intracellular ATP levels. The intracellular ATP content of hepatocytes with exogenous NADH was significantly higher than that of the normal group without added NADH, whereas pretreatment with the 3-NPA followed by NADH addition resulted in a decrease in intracellular ATP content. The ATP content of HIRI hepatocytes decreased significantly, and the decrease of ATP content in HIRI hepatocytes could also be reversed by adding exogenous NADH (Figure 7G). Therefore, the above results indicated that a decrease of mitochondrial NADH content in hepatocytes results in a significant reduction of mitochondrial and intracellular ATP levels during HIRI.

### Potential Cascade Signaling Pathways of Intracellular O_2_•^–^—SDH—Mito NADH—Mito ATP—Intracellular ATP in the HIRI Process

Based on above experiments, the operation of the intracellular O_2_^•^^–^—SDH—Mito NADH—Mito ATP—intracellular ATP cascade signaling pathways in the process of HIRI was implied for the first time (Figure 7H). In general, excessive accumulation of O_2_^•^^–^ occurred in hepatocytes in the case of HIRI. Excess O_2_^•^^–^ inactivated SDH, an important hub of oxidative phosphorylation and the TCA cycle. The inactivation of SDH resulted in a decrease of mitochondrial NADH content in hepatocytes, thereby affecting the synthesis of mitochondrial ATP and ultimately leading to the reduction of intracellular ATP content. The signaling pathways fully confirmed the important role of O_2_^•^^–^ and ATP in HIRI, providing a foundation for further studies of interlinked active signaling molecules in HIRI.

## Conclusions

To achieve the simultaneous detection of O_2_^•^^–^ and ATP in HIRI, a dual-color and dual-reversible molecular platform (UDP) with high sensitivity and excellent selectivity in response to O_2_^•^^–^ and ATP was developed. UDP can react with O_2_^•^^–^ and ATP synchronously and reversibly, and independently monitor fluorescence in the blue and red channels without interference from spectral crosstalk. The excellent performance of UDP in response to O_2_^•^^–^ and ATP in vitro enabled the simultaneous imaging and dynamic monitoring of endogenous O_2_^•^^–^ and ATP in hepatocytes. UDP was then used to visualize in situ O_2_^•^^–^ and ATP dynamics in hepatocytes during HIRI and drug treatment in real-time via the well-separated blue and red signals. More importantly, UDP realized the visualization of O_2_^•^^–^ and ATP in mouse livers during HIRI. Finally, the intracellular O_2_^•^^–^—SDH—Mito NADH—Mito ATP—intracellular ATP cascade-mediated signaling pathways in HIRI was uncovered for the first time. In summary, this research provides a fluorescent probe able to investigate the correlation and synergy between O_2_^•^^–^ and ATP in HIRI and can provide insight into the relationships of interlinked active molecules in the progression of HIRI.

## Supporting Information Available

Experimental data including experimental details, synthesis of UDP, photophysical properties, and fluorescence imaging of hepatocytes (PDF)
