# Supplementary material for: Unveiling the Crucial Roles of O2•– and ATP in Hepatic Ischemia–Reperfusion Injury Using Dual-Color/Reversible Fluorescence Imaging
Source: J Am Chem Soc. 2023 Sep 1;145(36):19662–75. doi: 10.1021/jacs.3c04303 (PMC10510312; doi:10.1021/jacs.3c04303)
Supplement: Supplementary file 1 — ja3c04303_si_001.pdf [file ja3c04303_si_001.pdf]

Supporting Information

Unveiling the Crucial Roles of $O_2^{\bullet-}$ and ATP in Hepatic Ischemia−Reperfusion Injury Using Dual-Color/Reversible Fluorescence Imaging

Jihong Liu,[a] Wen Zhang,[a]* Xin Wang,[a] Qi Ding,[a] Chuanchen Wu,[a] Wei Zhang,[a] Luling Wu,[ac]* Tony D. James,[acd]* Ping Li,[a]* and Bo Tang[ab]*

a. College of Chemistry, Chemical Engineering and Materials Science, Key Laboratory of Molecular and Nano Probes, Ministry of Education, Collaborative Innovation Center of Functionalized Probes for Chemical Imaging in Universities of Shandong, Institutes of Biomedical Sciences, Shandong Normal University, Jinan 250014, People's Republic of China.

b. Laoshan Laboratory, Qingdao 266237, People's Republic of China.

c. Department of Chemistry, University of Bath, Bath, BA2 7AY, UK.

d. School of Chemistry and Chemical Engineering, Henan Normal University, Xinxiang 453007, People's Republic of China.

Email: zhangwen@sdnu.edu.cn, wllcyl@126.com, t.d.james@bath.ac.uk, lip@sdnu.edu.cn, tangb@sdnu.edu.cn

Table of Contents

Experimental Procedures S4
Materials and instruments S4
Preparation of ROS/RNS S4
Fluorescence spectra of UDP toward $O_2^{\bullet-}$ in the presence of ATP S5
Fluorescence spectra of UDP toward ATP in the presence of $O_2^{\bullet-}$ S5
LOD calculation S5
HPLC Analysis S6
Cells culture S6
Cytotoxicity assays S6
Hepatic ischemia–reperfusion injury models in cells S6
Fluorescence imaging of $O_2^{\bullet-}$ and ATP under 2-ME stimulation S6
Fluorescence imaging of $O_2^{\bullet-}$ and ATP under oligomycin A stimulation S6
Fluorescence imaging of $O_2^{\bullet-}$ and ATP in hepatocytes during HIRI S7
Fluorescence imaging of intervention effect of HIRI drug S7
Fluorescence imaging of intracellular ROS during HIRI S7
Fluorescence imaging of ATP under 3-NPA stimulation S8
Reversible fluorescence imaging of $O_2^{\bullet-}$ fluctuations in hepatocytes S8
Reversible fluorescence imaging of ATP fluctuations in hepatocytes S8
Hepatic ischemia–reperfusion injury models in mice S8
Fluorescence imaging of $O_2^{\bullet-}$ and ATP in mice during HIRI S9
In vivo toxicity of UDP S9
Reversible fluorescence imaging of $O_2^{\bullet-}$ fluctuations in mice S9
Reversible fluorescence imaging of ATP fluctuations in mice S10
H&E staining of major organs in control group mice and HIRI group mice S10
BCA protein assays S10
ATP contents assays S10
Succinate dehydrogenase activity assays S11
Mitochondria extraction from hepatocytes S11
Mitochondrial NADH determination S12
Aspartate aminotransferase activity assays S12
Alanine aminotransferase activity assays S12
Tumor necrosis factor-α determination S13
Lactate dehydrogenase release determination S13
Proteomic analysis S13
Statistical analysis S14
Data availability S14
Results and Discussion S14
Synthesis of UDP S14
Scheme S1 .... S14
Figure S1. .... S15
Figure S2. .... S15
Figure S3. .... S16
Figure S4. .... S16

Figure S5. .... S17
Figure S6. .... S17
Figure S7. .... S18
Figure S8. .... S18
Figure S9. .... S19
Figure S10. .... S19
Figure S11. .... S20
Figure S12. .... S21
Figure S13. .... S22
Figure S14. .... S23
Figure S15. .... S24
Figure S16. .... S25
Figure S17. .... S26
Figure S18. .... S27
Figure S19. .... S27
Figure S20. .... S28
Figure S21. .... S28
Reference S29

Experimental Procedures

Materials and instruments

Caffeic acid was purchased from Shanghai Aladdin Biochemical Technology Co., Ltd. Rhodamine B was purchased from Shanghai Macklin Biochemical Co., Ltd. Diethylenetriamine was purchased from Shanghai Macklin Biochemical Co., Ltd. *N,N'*-dicyclohexylcarbodiimide and 4-dimethylaminopyridine were purchased from Shanghai Aladdin Biochemical Technology Co., Ltd. Adenosine 5'-triphosphate (ATP) disodium purchased from Bidepharm was used to represent ATP for all solution experiments. Apyrase was obtained from Shanghai yuanye Bio-Technology Co., Ltd. 2-Methoxyestradiol (2-ME) was purchased from Shanghai Aladdin Biochemical Technology Co., Ltd. Tiron was obtained from Sinopharm Chemical Reagent Co., Ltd. Oligomycin A was obtained from MedChemExpress. ATP disodium salt was purchased from MedChemExpress for the exogenous addition of ATP in the cell work. *N*-acetyl-L-cysteine were purchased from Shanghai Aladdin Biochemical Technology Co., Ltd. Reactive Oxygen Species Assay Kit (S0033S, Beyotime) were used. Bicinchoninic acid (BCA) Protein Assay Kit was purchased from GenStar. ATP Contents Assay Kit and Succinate Dehydrogenase Activity Assay Kit were obtained from Nanjing Jiancheng Bioengineering Institute. Mitochondrial Isolation Kit (CS0201) was obtained from Biotopped. NAD^+/NADH Assay Kit with WST-8 (S0175) was purchased from Beyotime. 3-Nitropropanoic acid and NADH disodium salt were purchased from MedChemExpress. Aspartate Aminotransferase (AST) Activity Assay Kit and Alanine Aminotransferase (ALT) Activity Assay Kit were purchased from Elabscience. Tumor Necrosis Factor-α (TNF-α) ELISA kit was purchased from 4A Biotech Co., Ltd. Lactate Dehydrogenase (LDH) release assay kit was obtained from Beyotime.

Absorption spectra were recorded on a UV-Visible spectrophotometer (Evolution 220, Thermo Scientific). Fluorescence spectra were obtained with a Hitachi F-4700 fluorescence spectrophotometer. CCK-8 assay was performed using a Triturus microplate reader. Confocal imaging was performed on Leica SP8 high-resolution fluorescence microscope. The mass spectra were obtained using the Bruker Maxis ultra-high-resolution-TOF MS system. Mice liver slices of 100 μm thickness were obtained with cryostat (Leica CM1950). Proteomic analysis was performed by Q Exactive (Thermo Fisher) through LC-MS/MS. Hematoxylin-eosin (H&E) staining images in Figure 5 were obtained using an optical microscope (Leica, DM2500). H&E staining images in Figure S17 were obtained using an optical microscope (Nikon, Eclipse Ci-L). ^{1}H NMR spectra were obtained at 400 MHz using Bruker NMR spectrometers, and ^{13}C NMR spectra were recorded at 100 MHz. HPLC analysis was carried out on a Shimadzu LC-16 system equipped with SPD-16 UV−vis detector.

Preparation of ROS/RNS

$O_2^{\bullet-}$

$O_2^{\bullet-}$ was produced from KO_2 in dry DMSO by an ultrasonic method. The concentration of $O_2^{\bullet-}$ was determined from the absorption at 250 nm (ε = 2682 M^{-1} cm^{-1}).

$ONOO^-$

0.6 M $NaNO_2$, 0.6 M HC1 and 0.7 M H_2O_2 were added simultaneously to a 3 M NaOH solution at 0 ºC. The concentration of $ONOO^-$ was determined using extinction coefficient of 1670 M^{-1} cm^{-1} at 302 nm in 0.1 M NaOH (aq.).

H_2O_2

H_2O_2 solutions were accessed by dilution of 30% hydrogen peroxide aqueous solution, the concentration was determined from the absorption at 240 nm (ε = 43.6 M^{-1} cm^{-1}).

TBHP

TBHP solutions were accessed by dilution of 70 % *tert*-butyl hydroperoxide aqueous solution.

$^•OH$

$^•OH$ (hydroxyl radical) was generated by the Fenton reaction of $FeCl_2$ with H_2O_2 (1:6) in deionized water.[1]

NO

NO (Nitric oxide) was obtained from a stock solution prepared by sodium nitroprusside (SNP).[2] Briefly, NO was prepared in the following manner: 4 mL of 10 mM SNP was dissolved in PBS buffer solutions (pH = 7.4) under light irradiation for 30 min. Then, the concentrations of NO release were determined by the Griess method reported previously (NO concentration assay kit, Beyotime).[3,4] 50 μL of Griess Reagent I and 50 μL of Griess Reagent II were added to 50 μL of SNP solutions, and then the mixture absorbance was measured at 540 nm. The concentrations of NO were calculated the standard curve of sodium nitrite standards.

$^1O_2$

$^1O_2$ (Singlet oxygen) was prepared by the reaction of 10 mM of NaClO and 10 mM of H_2O_2 in PBS buffer solutions (pH = 7.4).[5-7]

Fluorescence spectra of UDP toward $O_2^{•-}$ in the presence of ATP

A solution of ATP (22 mM) was added into UDP (25 μM), and the fluorescence spectrum was recorded after 25 min. Then various concentrations of $O_2^{•-}$ (0–65 μM) were added to the solution, and each line of the fluorescence spectra in $O_2^{•-}$ channel was recorded after 5 min. λ_{ex} = 380 nm.

Fluorescence spectra of UDP toward ATP in the presence of $O_2^{•-}$

A solution of $O_2^{•-}$ (65 μM) was added into UDP (25 μM), and the fluorescence spectrum was recorded after 5 min. Then various concentrations of ATP (0–22 mM) were added to the solution, and each line of the fluorescence spectra in ATP channel was recorded after 25 min. λ_{ex} = 520 nm.

LOD calculation

The limit of detection (LOD) was calculated using the well-established method (LOD = 3σ/K), where K is slope of the calibration curve, σ represents is the standard deviation of the blank sample (11 times) of F_{588} (for ATP) or F_{470} (for $O_2^{•-}$) for UDP without addition of ATP or $O_2^{•-}$.[8] (Note F_{588} and F_{470} refer to the Fluorescence (F) emission wavelength peaks at 588 and 470 nm).

HPLC Analysis

For HPLC analysis, deionized water was used as eluent A and methanol as eluent B. HPLC conditions: 14% of A, 86% of B. The injection volume was 10 μL. The parameters of the HPLC-MS analytical column used were C18-WR, 5 μm, 4.6 mm × 150 mm (GL Sciences). The purity of UDP was calculated to be 98% as determined by HPLC analysis. Purity data was calculated based on the integration in the HPLC trace at 254 nm. The flow rate is 0.7 mL/min.

Cells culture

Human hepatocytes (HL-7702) were purchased from the Cell Bank of the Chinese Academy of Sciences (Shanghai, China). Hepatocytes were cultured in high-glucose DMEM supplemented with 10 % fetal bovine serum, 1 % penicillin and 1 % streptomycin (w v^{-1}) at 37 °C in a 5 % CO_2/95 % air MCO-15AC incubator (SANYO, Tokyo, Japan).

Cytotoxicity assays

Cell Counting Kit-8 (CCK-8) assays were carried out to evaluate the toxicity of UDP. Hepatocytes (10^6 cells mL^{-1}) were seeded into 96-well microtiter plates with total volumes of 200 μL $well^{-1}$. After 24 h of incubation, various concentrations of UDP (0 M, 1×10^{-9} M, 1×10^{-8} M, 1×10^{-7} M, 1×10^{-6} M, 1×10^{-5} M, 1×10^{-4} M and 1×10^{-3} M) were added, and the hepatocytes were cultured for another 24 h. Afterwards, 10 μL of CCK-8 solution was added to each well. After 4 h of incubation, the absorbance at 450 nm was measured using a Triturus microplate reader.

Hepatic ischemia−reperfusion injury models in cells

Hepatic ischemia−reperfusion injury (HIRI) cell models were established by oxygen-glucose-serum deprivation/reperfusion. For the phase of ischemia, HL-7702 cells were cultured in DMEM (without glucose and serum) and deoxygenated sodium dithionite (0.5 mM) for 20 min or 40 min ischemia. For the phase of subsequent reperfusion, these cells were incubated with high glucose and serum DMEM (standard DMEM) in a 5 % CO_2 and 95 % O_2 atmosphere for 20 min or 40 min reperfusion after 40 min ischemia.

Fluorescence imaging of $O_2^{\bullet-}$ and ATP under 2-ME stimulation

HL-7702 cells were divided into three groups. The control group cells were stained with UDP (40 μM) for 20 min. The 2-ME stimulated group cells were incubated with 2-ME (3.0 μg/mL) for 1 h, then stained with UDP (40 μM) for 20 min. The Tiron + 2-ME stimulated group cells were pretreated with Tiron (10 μM) for 1 h, followed by adding 2-ME (3.0 μg/mL) for 1 h and then stained with UDP (40 μM) for 20 min. The cell culture medium of each group was removed, and all cells were washed with 1.0 mL of PBS three times before fluorescence imaging. One-photon confocal photographs were taken using a Leica SP8 high-resolution fluorescence microscope equipped with the Leica Application Suite X software package.

Fluorescence imaging of $O_2^{\bullet-}$ and ATP under oligomycin A stimulation

The control group cells were stained with UDP (40 μM) for 20 min. The oligomycin A group cells were

incubated with oligomycin A (50 μM) for 1 h, then stained with UDP (40 μM) for 20 min. The oligomycin A + ATP stimulated group cells were incubated with oligomycin A (50 μM) for 1 h prior to treating with ATP (10 mM) for 1 h. They were then stained with UDP (40 μM) for 20 min. The cell culture medium of each group was removed, and all cells were washed with 1.0 mL of PBS three times before fluorescence imaging. One-photon confocal photographs were taken imaged by Leica SP8 high-resolution fluorescence microscope and Leica Application Suite X software.

Fluorescence imaging of $O_2^{\bullet-}$ and ATP in hepatocytes during HIRI

In order to perform fluorescence imaging of $O_2^{\bullet-}$ and ATP fluctuation during the process of HIRI, HL-7702 cells were divided into five groups. Control group hepatocytes were cultured with high glucose and serum DMEM in a 5 % CO_2 and 95 % O_2 atmosphere. The 20 min of ischemia group hepatocytes were cultured in DMEM (without glucose and serum) and deoxygenated sodium dithionite (0.5 mM) for 20 min. The 40 min of ischemia group hepatocytes were cultured in DMEM (without glucose and serum) and deoxygenated sodium dithionite (0.5 mM) for 40 min. For 40 min of ischemia followed by 20 min of reperfusion group, hepatocytes were cultured in DMEM (without glucose and serum) and deoxygenated sodium dithionite (0.5 mM) for 40 min. Afterwards, cells were incubated with high glucose and serum DMEM in a 5 % CO_2 and 95 % O_2 atmosphere for 20 min. For 40 min of ischemia followed by 40 min of reperfusion group, hepatocytes were cultured in DMEM (without glucose and serum) and deoxygenated sodium dithionite (0.5 mM) for 40 min. Afterwards, cells were incubated with high glucose and serum DMEM in a 5 % CO_2 and 95 % O_2 atmosphere for 40 min. All the groups were treated with 40 μM UDP for 20 min before confocal imaging.

Fluorescence imaging of intervention effect of HIRI drug

To investigate injury remediation, HL-7702 cells were pretreated with 0.5 mM or 1 mM NAC for 1 h and then cultured with DMEM (without glucose and serum) and deoxygenated sodium dithionite (0.5 mM) for 40 min, followed by incubation with standard DMEM in a 5 % CO_2 and 95 % O_2 atmosphere for 40 min. All the groups were treated with 40 μM UDP for 20 min before confocal imaging.

Fluorescence imaging of intracellular ROS during HIRI

HL-7702 cells were divided into five groups. The control group cells were placed under normal culture conditions. The 20 min of ischemia group hepatocytes were cultured in DMEM (without glucose and serum) and deoxygenated sodium dithionite (0.5 mM) for 20 min. The 40 min of ischemia group hepatocytes were cultured in DMEM (without glucose and serum) and deoxygenated sodium dithionite (0.5 mM) for 40 min. For 40 min of ischemia followed by 20 min of reperfusion group, hepatocytes were cultured in DMEM (without glucose and serum) and deoxygenated sodium dithionite (0.5 mM) for 40 min. Afterwards, cells were incubated with high glucose and serum DMEM in a 5 % CO_2 and 95 % O_2 atmosphere for 20 min. For 40 min of ischemia followed by 40 min of reperfusion group, hepatocytes were cultured in DMEM (without glucose and serum) and deoxygenated sodium dithionite (0.5 mM) for 40 min. Afterwards, cells were incubated with high glucose and serum DMEM in a 5 % CO_2 and 95 % O_2 atmosphere for 40 min. All the group cells were co-stained with DCFH-DA (10 μM) and Hoechst 33342 (1 μg/mL) for 20 min before confocal imaging.

Fluorescence imaging of ATP under 3-NPA stimulation

The control group cells were stained with UDP (40 μM) for 20 min. The 3-NPA & Control group cells were incubated with 3-NPA (3 mM) for 3 h, then stained with UDP (40 μM) for 20 min. The HIRI group cells were cultured in DMEM (without glucose and serum) and deoxygenated sodium dithionite (0.5 mM) for 40 min. Afterwards, HIRI cells were incubated with high glucose and serum DMEM in a 5 % CO_2 and 95 % O_2 atmosphere for 40 min. They were then stained with UDP (40 μM) for 20 min. The 3-NPA & HIRI group cells were pretreated with 3-NPA (3 mM) for 3 h, cultured in DMEM (without glucose and serum) and deoxygenated sodium dithionite (0.5 mM) for 40 min, and incubated with high glucose and serum DMEM in a 5 % CO_2 and 95 % O_2 atmosphere for 40 min. They were then stained with UDP (40 μM) for 20 min. The cell culture medium of each group was removed, and all cells were washed with 1.0 mL of PBS three times before fluorescence imaging. One-photon confocal photographs were taken imaged by Leica SP8 high-resolution fluorescence microscope and Leica Application Suite X software.

Reversible fluorescence imaging of $O_2^{\bullet-}$ fluctuations in hepatocytes

The control group cells were stained with UDP (40 μM) for 20 min. Afterwards, 2-ME (3 μg/mL) was added in control cells. After the incubation of 2-ME for 30 min, 1 mM of GSH was added in the cells for 30 min. Then, 3 μg/mL of 2-ME was added in the hepatocytes and incubated for 30 min. Finally, cells were treated with1 mM of GSH for 30 min. One-photon confocal photographs were taken imaged by Leica SP8 high-resolution fluorescence microscope and Leica Application Suite X software.

Reversible fluorescence imaging of ATP fluctuations in hepatocytes

The control group cells were stained with UDP (40 μM) for 20 min. Afterwards, ATP (10 mM) was added in control cells. After the incubation of ATP for 30 min, 1 U/N of apyrase was added in the cells for 30 min. Then, 10 mM of ATP was added in the hepatocytes and incubated for 30 min. Finally, cells were treated with 1 U/N of apyrase for 30 min. One-photon confocal photographs were taken imaged by Leica SP8 high-resolution fluorescence microscope and Leica Application Suite X software.

Hepatic ischemia−reperfusion injury models in mice

Six-week-old C57 mice (males) were used. A HIRI mouse model was established by simulating liver surgery. The mice in HIRI group were given a laparotomy to expose the liver, and hepatic ischemia was induced by clamping the portal vein and hepatic artery of the median and the left lateral lobes of the liver with a microvessel clip for 20 min or 40 min, which induced partial (70 %) liver ischemia. Subsequently, the vascular clamp was opened for 20 min or 40 min reperfusion after 40 min ischemia. For normal group, the liver was exposed as a control. During the laparotomy surgery, the mice were anesthetized by inhaling isoflurane. All animal experiment methods were approved by Animal experiment ethical review committee of Shandong Normal University (Application number: AEECSDNU2023040).

Fluorescence imaging of $O_2^{\bullet-}$ and ATP in mice during HIRI

Mice were randomly divided into five group. The mice in the control group were subjected to a laparotomy and the livers were exposed. The mice in 20 min of ischemia group underwent a laparotomy surgery and the portal vein and hepatic artery of the median and the left lateral lobes of the liver were clamped with a microvessel clip for 20 min. The mice in 40 min of ischemia group underwent a laparotomy surgery and the portal vein and hepatic artery of the median and the left lateral lobes of the liver were clamped with a microvessel clip for 40 min. For 40 min of ischemia followed by 20 min of reperfusion group, the portal vein and hepatic artery of the median and the left lateral lobes of the liver were clamped with a microvessel clip for 40 min. Subsequently, the vascular clamp was opened for 20 min reperfusion. For 40 min of ischemia followed by 40 min of reperfusion group, the portal vein and hepatic artery of the median and the left lateral lobes of the liver were clamped with a microvessel clip for 40 min. Subsequently, the vascular clamp was opened for 40 min reperfusion. Afterwards, UDP with a dose of 100 μM was intravenously injected into all groups of mice through the tail vein, after 20 min, the mice were sacrificed and the livers of all groups were sectioned using cryostat (Leica CM1950). Finally, the sections were imaged through Leica SP8 high-resolution fluorescence microscope.

***In vivo* toxicity of UDP**

The C57 mice were divided into three groups consisting of control group, 100 μM of UDP-treated group and 1 mM UDP-treated group. The control group were intraperitoneally (i. p.) injected with saline (0.9 % NaCl) solution every day for two weeks. The UDP-treated group were intraperitoneally administrated UDP for two weeks with experimental concentration of 100 μM or 1 mM. The body weights of three groups of mice were recorded every day for two weeks. After intraperitoneal injection with saline or UDP for two weeks, hematoxylin and eosin (H&E) staining of major organ tissues (liver, spleen, lung, heart, and kidney) was conducted to identify the histological changes. The tissues samples were fixed with 4% paraformaldehyde, and dehydrated, embedded, sectioned and stained by hematoxylin and eosin. Finally, the sections were imaged using an optical microscope (Nikon, Eclipse Ci-L).

Reversible fluorescence imaging of $O_2^{\bullet-}$ fluctuations in mice

The C57 mice were divided into five groups. The control group mice were intraperitoneally injected with UDP (100 μM) for 20 min. The 2-ME group of mice were intraperitoneally injected with UDP (100 μM) for 20 min, followed by the injection of 2-ME (15 μg/mL) for 20 min. The GSH group of mice were intraperitoneally injected with UDP (100 μM) for 20 min and 2-ME (15 μg/mL) for the next 20 min, followed by the injection of GSH (5 mM) for 20 min. Another 2-ME group of mice were intraperitoneally injected with UDP (100 μM) for 20 min and 2-ME (15 μg/mL) for next 20 min, followed by the injection of GSH (5 mM) for 20 min and the injection of 2-ME (15 μg/mL) for 20 min. Another GSH group of mice were intraperitoneally injected with UDP (100 μM) for 20 min and 2-ME (15 μg/mL) for next 20 min, followed by the injection of GSH (5 mM) for 20 min, the injection of 2-ME (15 μg/mL) for 20 min and the final injection of GSH (5 mM) for 20 min. All groups of mice were sacrificed and the livers of all groups were sectioned using cryostat (Leica CM1950).

Finally, the liver sections were imaged through Leica SP8 high-resolution fluorescence microscope.

Reversible fluorescence imaging of ATP fluctuations in mice

The C57 mice were divided into five groups. The control group mice were intraperitoneally injected with UDP (100 μM) for 20 min. The ATP group of mice were intraperitoneally injected with UDP (100 μM) for 20 min, followed by the injection of ATP (30 mM) for 30 min. The apyrase group of mice were intraperitoneally injected with UDP (100 μM) for 20 min and ATP (30 mM) for next 30 min, followed by the injection of apyrase (3 U/N) for 30 min. Another ATP group of mice were intraperitoneally injected with UDP (100 μM) for 20 min and ATP (30 mM) for the next 30 min, followed by the injection of apyrase (3 U/N) for 30 min and the injection of ATP (30 mM) for 30 min. Another apyrase group of mice were intraperitoneally injected with UDP (100 μM) for 20 min and ATP (30 mM) for the next 30 min, followed by the injection of apyrase (3 U/N) for 30 min, the injection of ATP (30 mM) for 30 min and the final injection of apyrase (3 U/N) for 30 min. All groups of mice were sacrificed and the livers of all groups were sectioned using cryostat (Leica CM1950). Finally, the liver sections were imaged through Leica SP8 high-resolution fluorescence microscope.

H&E staining of major organs in control group mice and HIRI group mice

The C57 mice were divided into two groups consisting of control group and HIRI group. Both control group and HIRI group were intraperitoneally (i. p.) injected with 100 μM of UDP. After intraperitoneal injection with UDP, H&E staining of major organ tissues (liver, spleen, lung, heart, and kidney) was conducted to identify the histological changes. The tissues samples were fixed with 4% paraformaldehyde, and dehydrated, embedded, sectioned and stained by hematoxylin and eosin. Finally, the sections were imaged using an optical microscope (Leica, DM2500).

BCA protein assays

The BCA protein assays were carried out by BCA Protein Assay Kit (GenStar). The principle of BCA determination method is that Cu^{2+} can be reduced to Cu^{+} by protein under alkaline conditions, and Cu^{+} binds with BCA reagent to form purple complex. By measuring the absorbance of samples at 562 nm and comparing with the standard curve of reference samples, the protein concentration of the sample to be measured can be calculated. The hepatocytes samples under various treatment were collected and placed in ice-water bath for homogenate crushing. 20 μL of reference sample and hepatocytes samples were added in microplate, respectively. 200 μL of BCA working solution was added in each well and vibrate to mix sufficiently. The microplate was covered and incubated for 30 min at 37 °C. After cooling to room temperature, the absorbance at 562 nm was measured using a Triturus microplate reader. The protein concentrations of samples were determined according to the standard curve of reference sample and the dilution ratio of samples.

ATP contents assays

The ATP contents in hepatocytes per unit protein weight were calculated combined with BCA Protein Assay kit and ATP Contents Assay Kit (Nanjing Jiancheng Bioengineering Institute). The principle of ATP determination method is that creatine kinase catalyzes ATP and creatine to produce creatine phosphate. For

ATP measurement, the phosphomolybdic acid colorimetric method was used to detect the generation of creatine phosphate. The hepatocytes samples under various treatment were collected and placed in ice-water bath for homogenate crushing. The cell suspensions were heated in a boiling water bath for 10 min, vortex mixed for 1 min and centrifuged at 3,500 rpm for 10 min. To measure ATP concentrations, 30 μL supernatant, 100 μL substrate I, 200 μL substrate II and 30 μL accelerator were combined and incubated at 37 °C for 30 min. Then, 50 μL precipitant was added, and the mixture was centrifuged at 4,000 rpm for 5 min. A volume of 500 μL chromogenic fluids were combined with 300 μL supernatant and incubated at room temperature for 2 min. Finally, 500 μL termination fluids were added and incubated at room temperature for 5 min. A 200 μL volume of the mixture was added to a 96-well plate, and the absorbance values were measured at 636 nm on a Triturus microplate reader. The ATP concentrations were calculated as follows:

$$C_{ATP} = (A_{Sample} - A_{Control}) / (A_{Reference} - A_{Blank}) \times C_{Reference} \times N / Cpr$$

in which C_{ATP} is the concentration of ATP (nmol/mg protein), A_{Sample} is the absorbance of the sample, $A_{Control}$ was the absorbance of the control, $A_{Reference}$ is the absorbance of the reference, A_{Blank} is the absorbance of the blank, $C_{Reference}$ is the concentration of the reference (1000 μmol/L), N is the sample dilution ratio before the determination, Cpr is the concentration of homogenate protein.

Succinate dehydrogenase activity assays

SDH activity assays were performed using a SDH Activity Assay Kit (Nanjing Jiancheng Bioengineering Institute). The SDH activity per unit protein weight (U/mg protein) were calculated combined with BCA Protein Assay kit and SDH Activity Assay Kit. Flavin adenine dinucleotide (FAD) is the auxiliary group of the SDH catalyzed reaction, and FAD is reduced to FADH, coupled with the reduction of 2,6-dichlorophenol indigo (2, 6-DPIP). The reduction rate of 2, 6-DPIP is determined to calculate the activity of SDH. The working solution was placed at 37 °C for 10 min. 2.6 mL of working solution were added to 100 μL of the samples and mixed. At the moment, the time point was defined as 0 min. 5 seconds later, the absorbance values at 600 nm were measured on a Triturus microplate reader. The absorbance values at 600 nm were measured again after 1 min and the difference between two absorbances was calculated. One unit of SDH activity was defined as a decrease of 0.01 in absorbance per minute at 600 nm in the reaction solutions. The SDH activity was calculated as follows:

$$A_{SDH} = [\Delta A / (0.01 \times T)] / (V_{Sample} \times Cpr)$$

in which A_{SDH} is the activity of SDH (U/mg protein), ΔA is the difference between absorbance at 5 s and absorbance at 1 min 5 s, T was reaction time (1 min), V_{Sample} is the volume of homogenate, Cpr is the concentration of homogenate protein.

Mitochondria extraction from hepatocytes

The mitochondria extraction was performed using a Mitochondria Extraction Kit (CS0201, Bjbalb, China). Hepatocytes under various treatment were trypsinized and centrifuged for 10 min at 1000 rpm. The cells were resuspended in PBS buffer and centrifuged for 10 min at 1000 rpm. The collected cells were added in 1 mL of reagent A and 10 μL of reagent D. Dounce homogenizer was used to homogenate many times in ice-bath. The

homogenate was centrifuged for 5 min at 600 × g at 4 °C and liquid supernatant was collected. Then the liquid supernatant was centrifuged for 10 min at 11000 × g at 4 °C and sediment was collected. The resulted sediment was intact mitochondria.

Mitochondrial NADH determination

Experiments to determine the mitochondrial NADH changes in hepatocytes under various treatments were performed using the NAD^+/NADH assay kit with WST-8 (Beyotime) and BCA Protein Assay kit. Ethanol is oxidized to acetaldehyde by alcohol dehydrogenase, in which NAD^+ is reduced to NADH, the resulting NADH reduces 2-(2-methoxy-4-nitrophenyl)-3-(4-nitrophenyl)-5-(2,4-disulfophenyl)-2H-tetrazolium sodium salt (WST-8) to formazan with the action of 1-Methoxy-5-methylphenazinium Methyl Sulfate (1-mPMS). After heating at 60 °C for 0.5 h, the NAD^+ in the samples will decompose and only the NADH will remain. NADH reduces WST-8 to formazan, and the concentrations of formazan produced by the reaction is determined by colorimetry, and the concentrations of NADH in the samples can ultimately be determined. For NAD^+/NADH extraction, 200 μL of NAD^+/NADH extraction buffer was added in the above mitochondria samples and blow gently, followed by spinning at 12000×g (5 min, 4 °C) to get the supernatant as a test sample. A series of various concentration of NADH standards were prepared. 100 μL of samples to be tested were placed in centrifuge tubes and heated at 60 °C for 0.5 h in a water bath. Alcohol dehydrogenase working solutions were mixed with NADH standards and test samples, respectively. After incubating for 10 min at 37 °C in dark place, 10 μL of chromogenic solution was added and incubated for 30 min at 37 °C. Finally, absorbances at 450 nm were recorded. The concentrations of mitochondrial NADH were calculated according to standard curve of NADH standards.

Aspartate aminotransferase activity assays

Aspartate aminotransferase (AST) activity assays were performed using an AST Activity Assay Kit (Elabscience). AST catalyze the reaction between α-ketone glutaric acid and aspartic acid, generating glutamic acid and oxaloacetic acid. Oxaloacetic acid decarboxylates to pyruvic acid, and pyruvic acid and 2,4-dinitrobenzene hydrazine generate 2,4-dinitrobenzene hydrazone, which appears as reddish brown in alkaline solution. The AST activity per unit protein weight (IU/g protein) were calculated combined with BCA Protein Assay kit and AST Activity Assay Kit. 20 μL of substrate solution was added, followed by the addition of 5 μL of samples. The mixture of substrate solution and samples were mixed and placed at 37 °C for 30 min. Afterwards, 20 μL of chromogenic agent was added in the mixture and incubated at 37 °C for 20 min. 200 μL of Alkali reagent was added and mixed at room temperature for 15 min. The absorbance at 510 nm was measured. The levels of AST in samples were calculated according to standard curve.

Alanine aminotransferase activity assays

Alanine aminotransferase activity assays (ALT) activity assays were performed using an ALT Activity Assay Kit (Elabscience). ALT catalyzed the reaction between alanine and α-ketone glutaric acid, producing glutamic acid and pyruvic acid. 2,4-dinitrobenzene hydrazine was added to stop this reaction, generating pyruvate

phenylhydrazone which appears as reddish brown in alkaline conditions. The ALT activity per unit protein weight (IU/g protein) were calculated combined with BCA Protein Assay kit and ALT Activity Assay Kit. 20 μL of substrate solution was added, followed by the addition of 5 μL of samples. The mixture of substrate solution and samples were mixed and placed at 37 °C for 30 min. Afterwards, 20 μL of chromogenic agent was added in the mixture and incubated at 37 °C for 20 min. 200 μL of Alkali reagent was added and mixed at room temperature for 15 min. The absorbance at 510 nm was measured. The levels of ALT in samples were calculated according to standard curve.

Tumor necrosis factor-α determination

Tumor necrosis factor-α (TNF-α) determination were performed using a TNF-α ELISA kit (4A Biotech Co., Ltd). After established HIRI models in hepatocytes, the supernatants were then collected and assayed for TNF-α. A monoclonal antibody specific for TNF-α was coated onto 96-well microtiter plates provided. TNF-α present in the samples or standard and the monoclonal anti TNF-α antibody conjugated to biotin were simultaneously incubated in microtiter plates. Following incubation unbound TNF-α was removed during a wash step. Streptavidin-HRP was added and bound to the biotinylated anti TNF-α. After incubation and wash step a substrate solution reactive with HRP was added to the wells. The reaction was terminated by adding stop solution. A yellow product was formed in proportion to the amount of TNF-α present in the sample. The absorbance was measured at 450 nm. The levels of TNF-α in samples were calculated according to standard curve.

Lactate dehydrogenase release determination

Lactate dehydrogenase (LDH) release determination were performed using a LDH release assay kit (Beyotime). After established HIRI models in hepatocytes, the supernatants were then collected and assayed for LDH. Under the action of LDH, nicotinamide adenine dinucleotide (NAD^+) is reduced to NADH, which react with 2-p-iodophenyl-3-nitrophenyl tetrazolium chloride to produce formazan under the catalytic reaction of diaphorase. The absorbance at 490 nm exhibit linear increases with enhanced activity of LDH. Hepatocytes were seeded into a 96-well plate and divided into control group and HIRI group. After treatment, the cell culture medium was collected, and LDH activity was measured using an LDH assay kit. An LDH releasing agent was provided in the kit and served as the positive control of total LDH release. Absorbance was measured at 490 nm using a microplate reader.

Proteomic analysis

Succinate dehydrogenase (1.0 mg mL^{-1}) was incubated for 3 h at 37 °C in buffer (10 mM PBS, pH 7.4) supplemented with 100 μM $O_2^{\bullet-}$. The succinate dehydrogenase was then subjected to lyophilization and trypsin digestion. The peptides were isolated from the hydrolysate by solid-phase extraction on a C-18 column. Proteomic analysis was then performed through LC-MS/MS. The LC-MS/MS data were submitted to Mass spectrometry matching software (MASCOT) for analysis, to obtain the identification information of polypeptide.

Statistical analysis

All data are expressed as the mean ± S.D. The data under each condition were accumulated from at least three independent experiments. For each experiment, unless otherwise noted, n represents the number of individual biological replicates. For each biological replicate and for all *in vitro* and *ex vivo* studies, $n \geq 3$. The Student's t test was used for comparisons between two groups of experiments. Statistically significant P values are indicated in Figures and/or legends as ***$P < 0.001$, **$P < 0.01$.

Data availability

All relevant data that support the findings of this study are available from the corresponding author upon reasonable request.

Results and Discussion

Synthesis of UDP

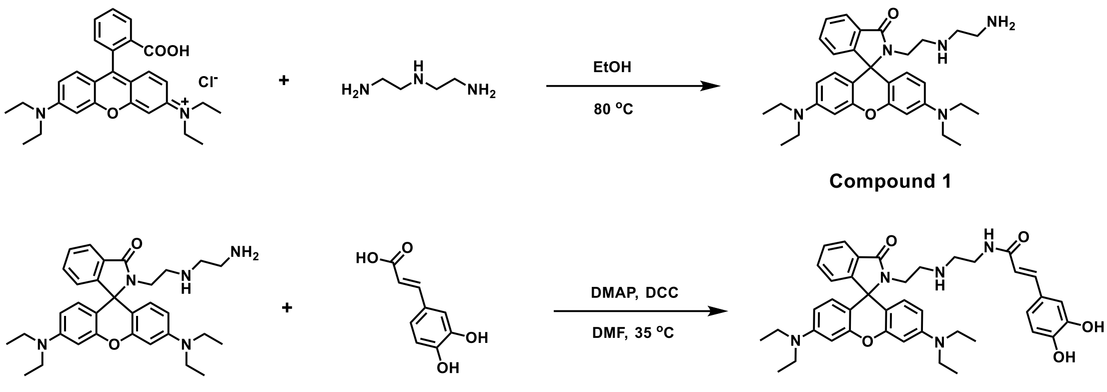

Scheme S1. The synthesis route of UDP.

Synthesis of Compound 1

Under a nitrogen atmosphere, rhodamine B (2.0 g, 4.2 mmol) was added to absolute ethanol (20 mL), followed by the addition of diethylenetriamine (2.0 mL, 18.4 mmol). The mixture was refluxed at 80 °C for 18 h, then allowed to cool down to room temperature. The crude product was subsequently purified by thin layer chromatography, eluting with dichloromethane/methanol (10:1, v/v) to afford compound 1 as a light-yellow solid (0.542 g, 1.03 mmol, 25%). HRMS (ES^+): calc. for $C_{32}H_{41}N_5O_2$ $[M+H]^+$ m/z 528.3333, found m/z 528.3255.

Synthesis of UDP

Caffeic acid (0.270 g, 1.5 mmol), triethylamine (168 μL, 1.2 mmol) were dissolved in extra dry *N,N*-dimethylformamide (10 mL), followed by the addition of 4-dimethylaminopyridine (0.147 g, 1.2 mmol) and *N,N'*-dicyclohexylcarbodiimide (0.248 g, 1.2 mmol) in ice-water bath. Then compound 1 (0.633 g, 1.2 mmol) was added in the above reaction solution and the entire mixture was refluxed at 35 °C for 24 h under a nitrogen atmosphere. The crude was subsequently purified by thin layer chromatography, eluting with

dichloromethane/methanol (5:1, v/v) to afford UDP as a dark-yellow solid (0.083 g, 0.12 mmol, 10%). HRMS (ES^+): calc. for $C_{41}H_{47}N_5O_5$ $[M+H]^+$ m/z 690.3650, found m/z 690.3744. ^{1}H NMR (400 MHz, DMSO-d_6): δ 7.79-7.77 (m, 1H), 7.52-7.47 (m, 2H), 7.17 (d, J = 16 Hz, 1H), 7.03-7.01 (m, 1H), 6.89 (s, 1H), 6.74-6.61 (m, 2H), 6.37-6.32 (m, 6H), 6.28 (d, J = 16 Hz, 1H), 3.31 (q, J = 8 Hz, 8H), 3.08-3.02 (m, 4H), 2.32 (t, J = 4 Hz, 2H), 2.18 (t, J = 8 Hz, 2H), 1.07 (t, J = 8 Hz, 12H). ^{13}C NMR (100 MHz, DMSO-d_6): δ 167.50, 166.68, 166.07, 153.85, 153.08, 148.84, 139.88, 133.05, 132.17, 131.99, 131.03, 129.12, 128.77, 128.69, 124.05, 122.72, 120.73, 116.09, 113.60, 108.62, 105.54, 97.70, 65.48, 64.46, 63.27, 48.82, 47.40, 44.15, 12.84.

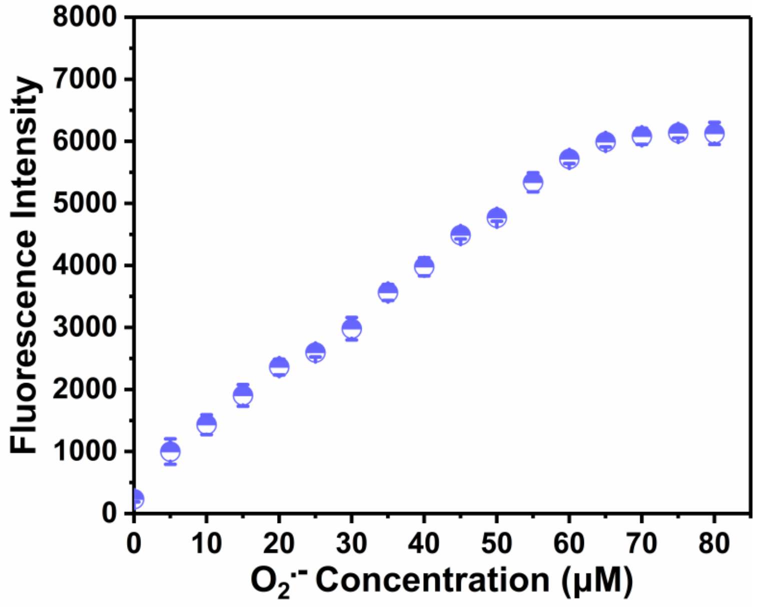

Figure S1. Emission at 470 nm of UDP (25 μM) as a function of increasing $O_2^{\bullet-}$ concentration (from 0 to 80 μM) in PBS buffer solution (10 mM, pH = 7.40). $\lambda_{ex/em}$ = 380/470 nm.

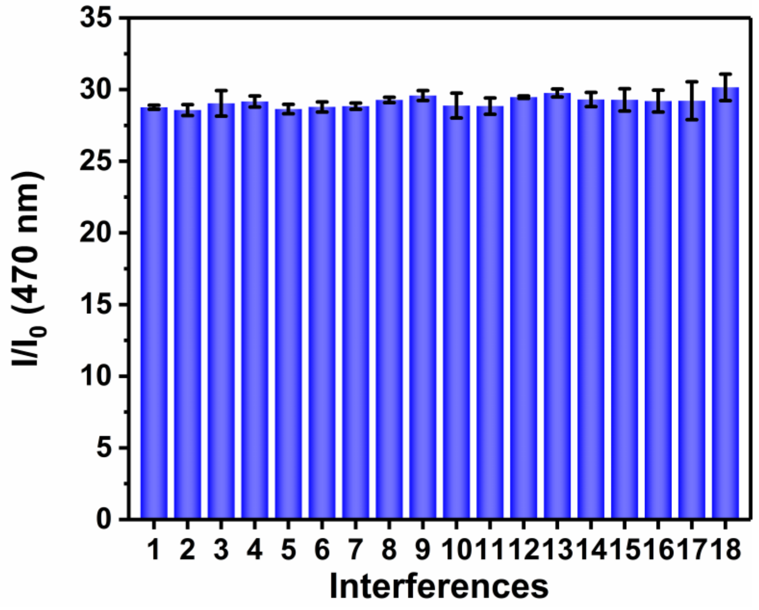

Figure S2. Blue fluorescence responses at 470 nm of a mixture of UDP (25 μM) and $O_2^{\bullet-}$ (65 μM) to various ROS, RNS and metal ions (1-18: Blank, 1 mM His, 1 mM Pro, 1 mM Arg, 10 mM K^+, 10 mM Na^+, 200 μM Ca^{2+}, 200 μM Mg^{2+}, 200 μM Cu^{2+}, 200 μM Fe^{3+}, 50 μM $NO^{\bullet}$, 100 μM $^{\bullet}OH$, 10 mM H_2O_2, 100 μM $^1O_2$, 100 μM TBHP, 100 μM $ROO^{\bullet}$, 100 μM NaClO, 25 μM $ONOO^-$) in PBS buffer solution (10 mM, pH = 7.40). $\lambda_{ex/em}$

= 380/470 nm.

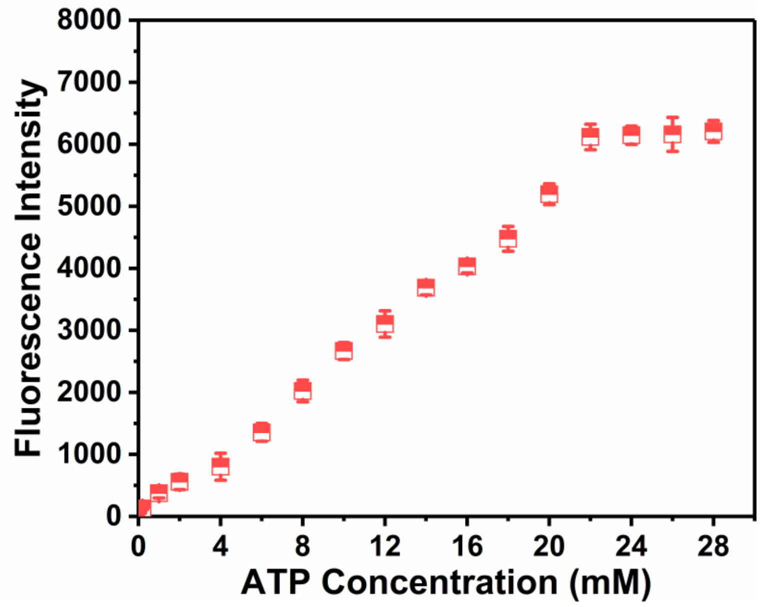

Figure S3. Emission at 588 nm of UDP (25 μM) as a function of increasing ATP concentration (from 0 to 28 mM) in PBS buffer solution (10 mM, pH = 7.40). $\lambda_{ex/em}$ = 520/588 nm.

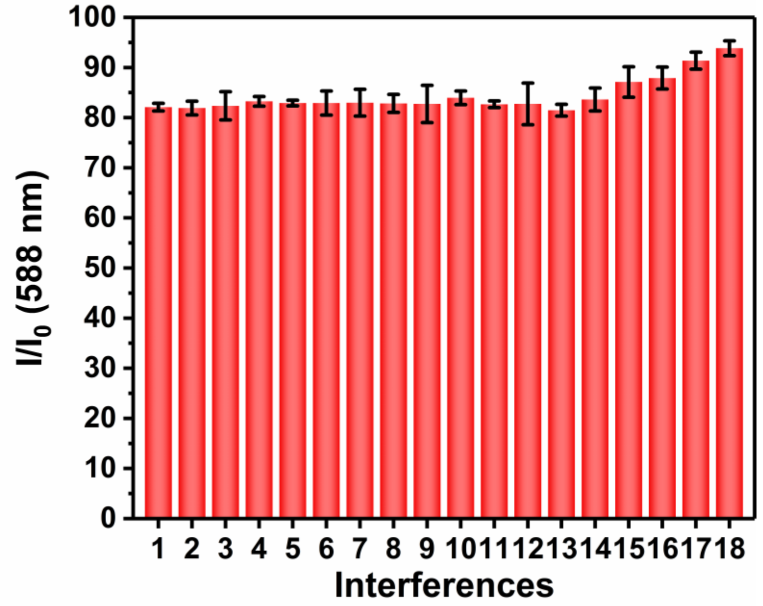

Figure S4. Red fluorescence responses at 588 nm of a mixture of UDP (25 μM) and ATP (10 mM) to various metal ions, anions, adenosine phosphates, and nucleoside triphosphates (1-18: Blank, 1 mM Cys, 1 mM GSH, 10 mM K^+, 10 mM Na^+, 200 μM Ca^{2+}, 200 μM Mg^{2+}, 200 μM Zn^{2+}, 10 mM PO_4^{3-}, 10 mM HPO_4^{2-}, 10 mM $H_2PO_4^-$, 10 mM SO_4^{2-}, 10 mM CO_3^{2-}, 10 mM UTP, 10 mM CTP, 10 mM GTP, 10 mM AMP, 10 mM ADP) in PBS buffer solution (10 mM, pH = 7.40). $\lambda_{ex/em}$ = 520/588 nm.

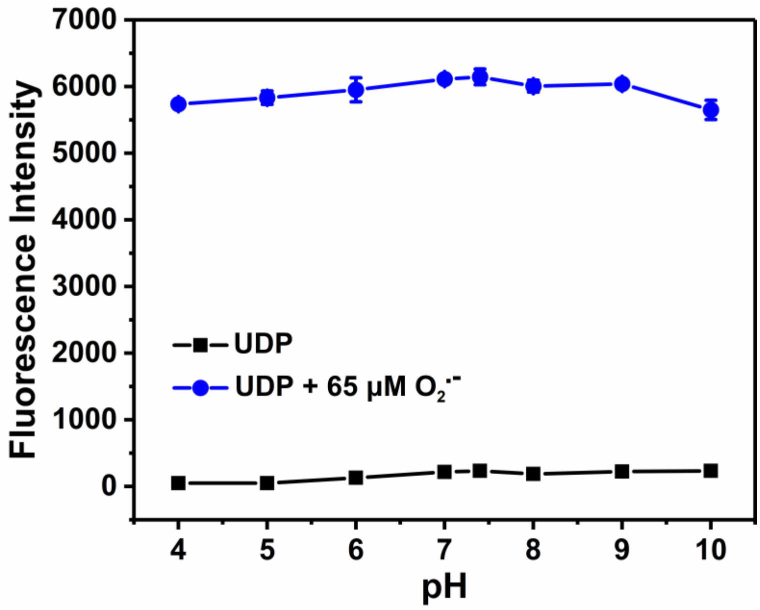

Figure S5. Fluorescence spectra of UDP (25 μM) in PBS buffer (10 mM, black squares) and after the addition of $O_2^{\bullet-}$ (65 μM, blue circles) at various pH's. $\lambda_{ex/em}$ = 380/470 nm.

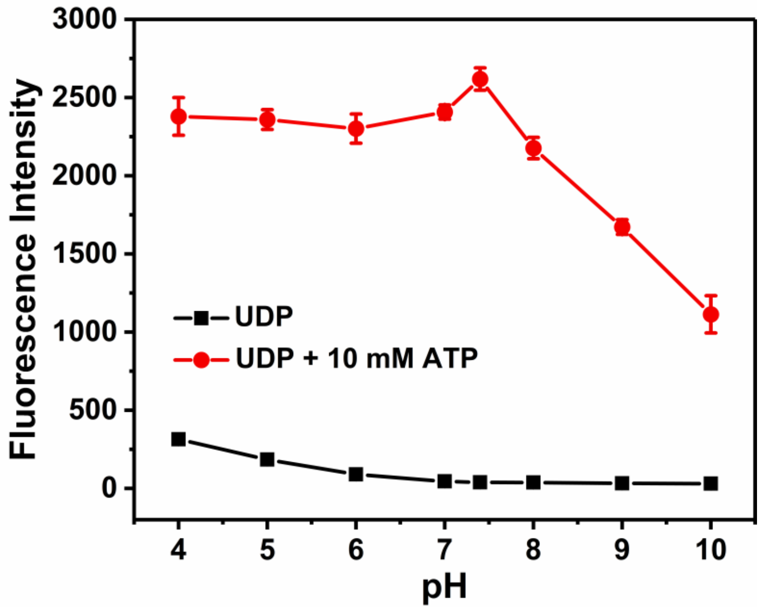

Figure S6. Fluorescence spectra of UDP (25 μM) in PBS buffer (10 mM, black squares) and after the addition of ATP (10 mM, red circles) at various pH's. $\lambda_{ex}/_{em}$ = 520/588 nm.

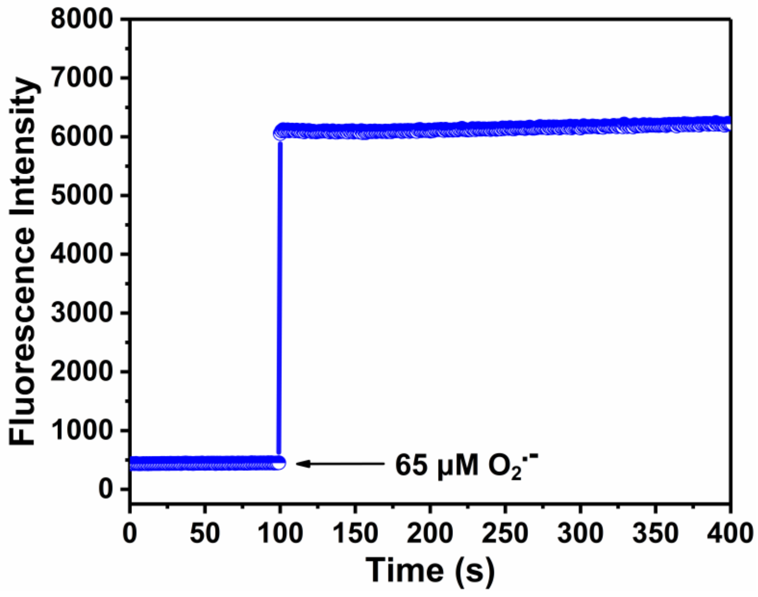

Figure S7. Emission at 470 nm of UDP (25 μM) upon the addition of $O_2^{\bullet-}$ (65 μM) in PBS buffer solution (10 mM, pH = 7.40). $\lambda_{ex}/_{em}$ = 380/470 nm.

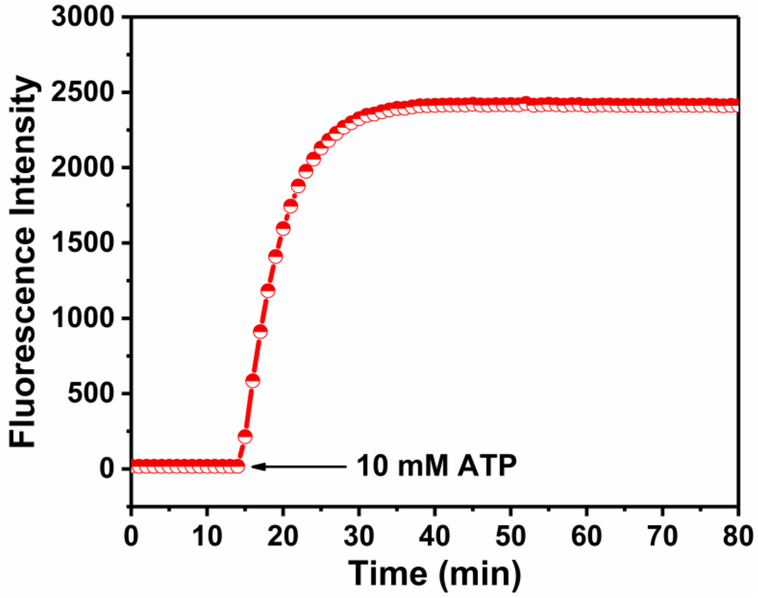

Figure S8. Emission at 588 nm of UDP (25 μM) upon the addition of ATP (10 mM) in PBS buffer solution (10 mM, pH = 7.40). $\lambda_{ex}/_{em}$ = 520/588 nm.

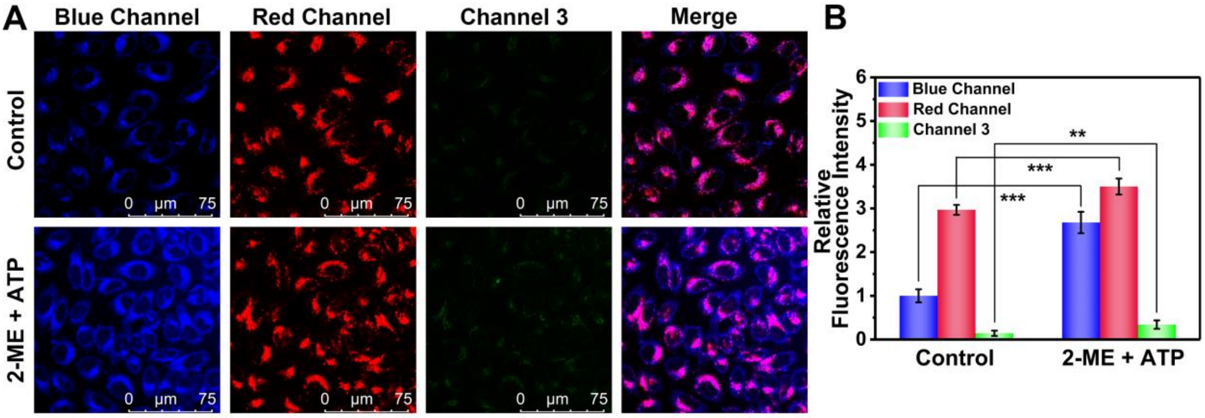

Figure S9. Confocal fluorescence imaging of $O_2^{\bullet-}$ and ATP fluctuations in hepatocytes stimulated by 2-ME and ATP. (A) Confocal fluorescence images of $O_2^{\bullet-}$ (blue channel, λ_{ex} = 405 nm, λ_{em} = 420-490 nm) and ATP (red channel, λ_{ex} = 514 nm, λ_{em} = 525-668 nm) in hepatocytes with UDP staining (40 μM, 20 min) after incubation of 2-ME (3 μg/mL, 1 h) and ATP (10 mM, 1 h). Channel 3 (λ_{ex} = 405 nm, λ_{em} = 525-668 nm). (B) Relative blue, red and green fluorescence intensity output of control group and 2-ME + ATP group. Note: The blue fluorescence intensity of control group was defined as 1.0. The data are expressed as the mean ± SD. **P < 0.01. ***P < 0.001. Similar results were obtained in five independent experiments.

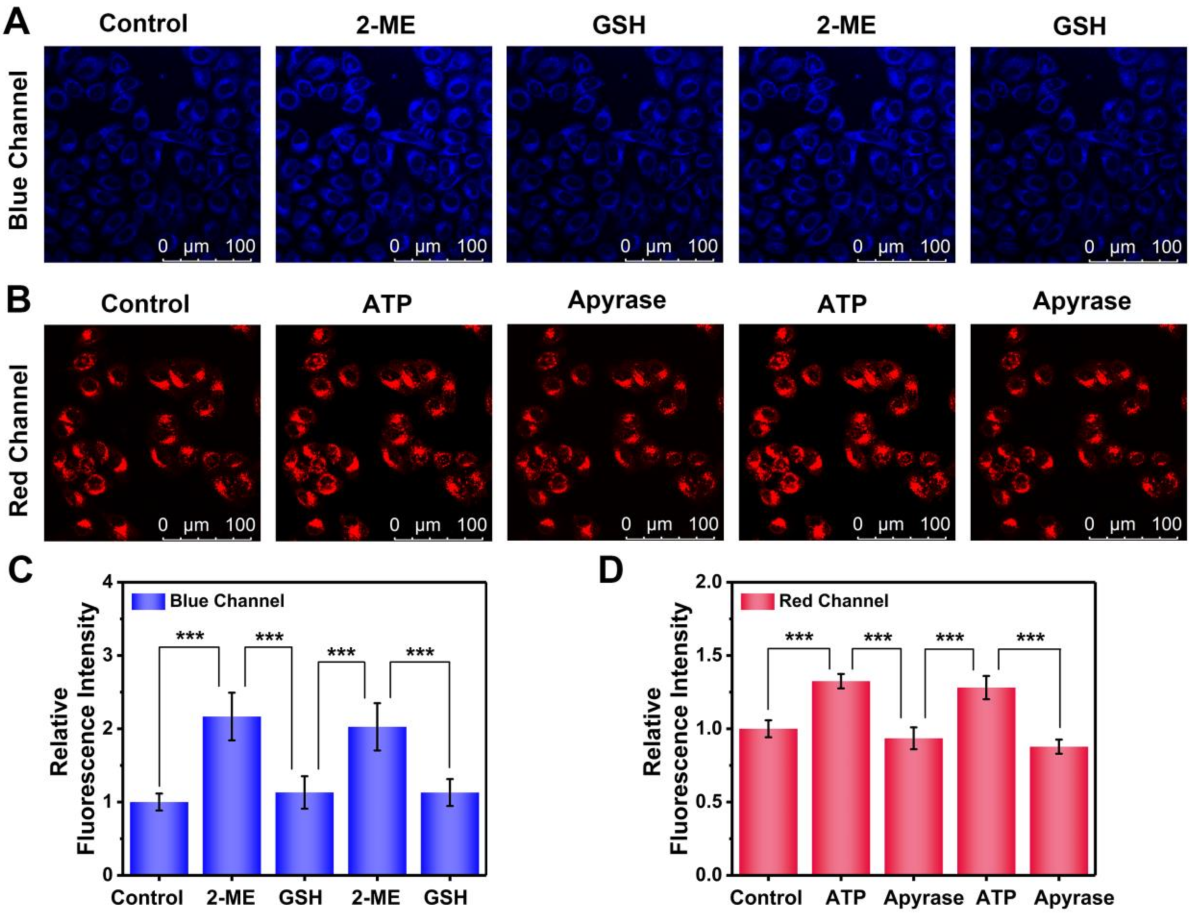

Figure S10. Reversible fluorescence imaging of $O_2^{\bullet-}$ and ATP fluctuations in hepatocytes. (A) Reversible blue fluorescence images of $O_2^{\bullet-}$ (λ_{ex} = 405 nm, λ_{em} = 420-490 nm) in hepatocytes with UDP staining (40 μM) after

incubation of 2-ME (3 μg/mL) and GSH (1 mM). (B) Reversible red fluorescence images of ATP (λ_{ex} = 514 nm, λ_{em} = 525-668 nm) in hepatocytes with UDP staining (40 μM) after incubation of ATP (10 mM) and apyrase (1 U/N). (C), (D) Relative blue and red fluorescence intensity output of (A), (B), respectively. Note: The blue and red fluorescence intensity of control group was defined as 1.0. The data are expressed as the mean ± SD. ***P < 0.001. Similar results were obtained in five independent experiments.

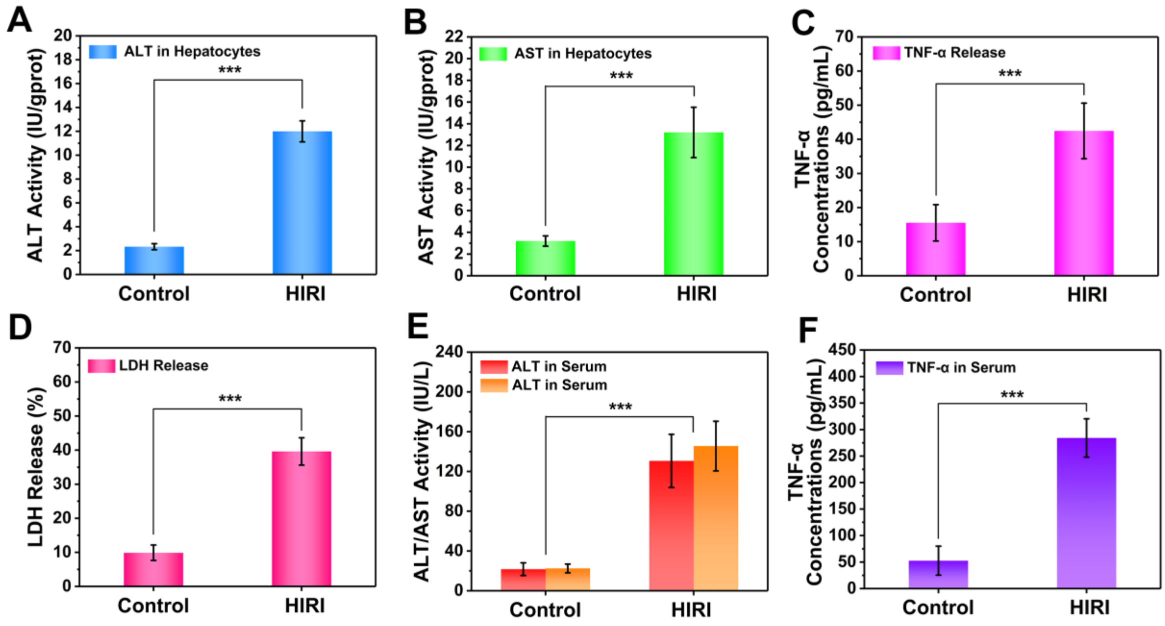

Figure S11. The measurement of hepatocyte injury markers in hepatocytes and mice. (A, B) ALT and AST activity assays in control hepatocytes and HIRI hepatocytes. (C) TNF-α concentrations in the cell supernatant of control hepatocytes and HIRI hepatocytes. (D) LDH release in the cell supernatant of control hepatocytes and HIRI hepatocytes. (E) ALT and AST activity assays in serum of control group mice and HIRI group mice. (F) TNF-α concentrations in the serum of control group mice and HIRI group mice.

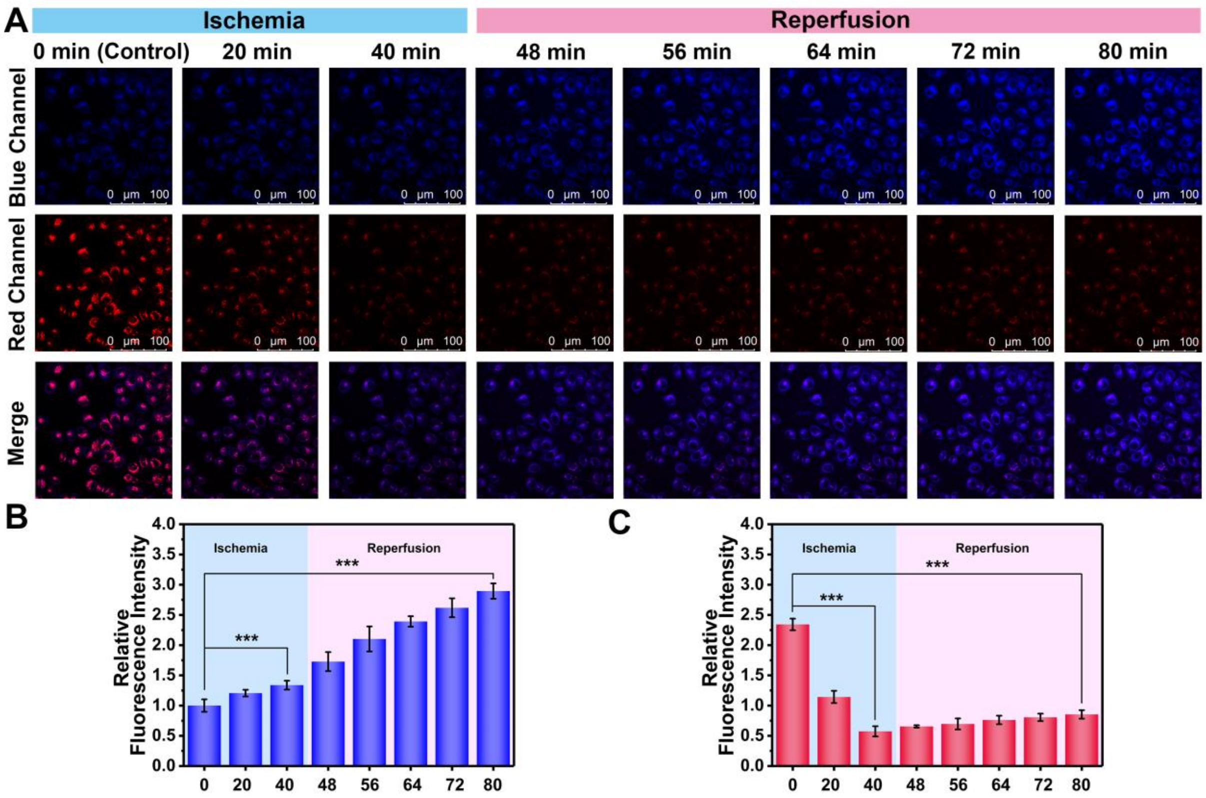

Figure S12. *In situ* visualization of $O_2^{\bullet-}$ and ATP dynamics in hepatocytes during the whole process of HIRI. (A) Fluorescence imaging of $O_2^{\bullet-}$ (blue channel, λ_{ex} = 405 nm, λ_{em} = 420-490 nm) and ATP (red channel, λ_{ex} = 514 nm, λ_{em} = 525-668 nm) by UDP (40 μM) in hepatocytes undergoing 0 min, 20 min or 40 min of ischemia and 8 min, 16 min, 24 min, 32 min or 40 min of reperfusion after 40 min of ischemia. (B, C) Relative blue and red fluorescence intensity output of (A). The blue fluorescence intensity of control group was defined as 1. The data are expressed as the mean ± SD. ***P < 0.001. Concordant results were obtained from five independent experiments.

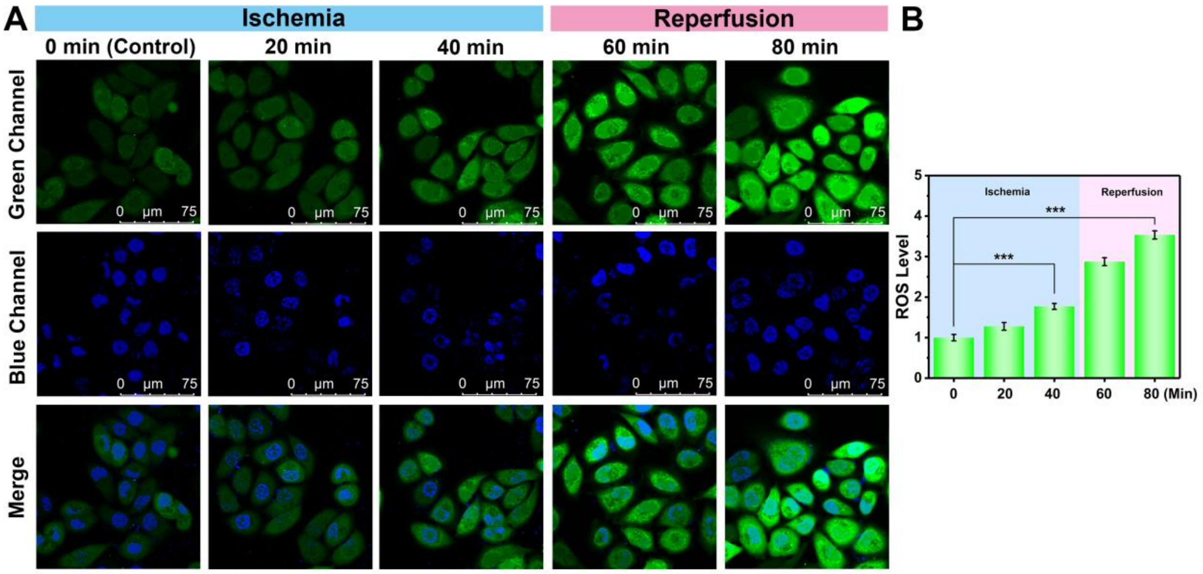

Figure S13. Confocal fluorescence imaging of ROS levels in hepatocytes during the whole process of HIRI. (A) Fluorescence imaging of ROS (green channel, λ_{ex} = 488 nm, λ_{em} = 505-540 nm) and nucleus (blue channel, λ_{ex} = 405 nm, λ_{em} = 420-500 nm) by DCFH-DA (10 μM) and Hoechst 33342 (1 μg/mL) in hepatocytes undergoing 0 min, 20 min or 40 min of ischemia and 20 min or 40 min of reperfusion after 40 min of ischemia. (B) Relative green fluorescence intensity output of (A). Note: The green fluorescence intensity of control group was defined as 1.0. The data are expressed as the mean ± SD. Similar results were obtained in five independent experiments.

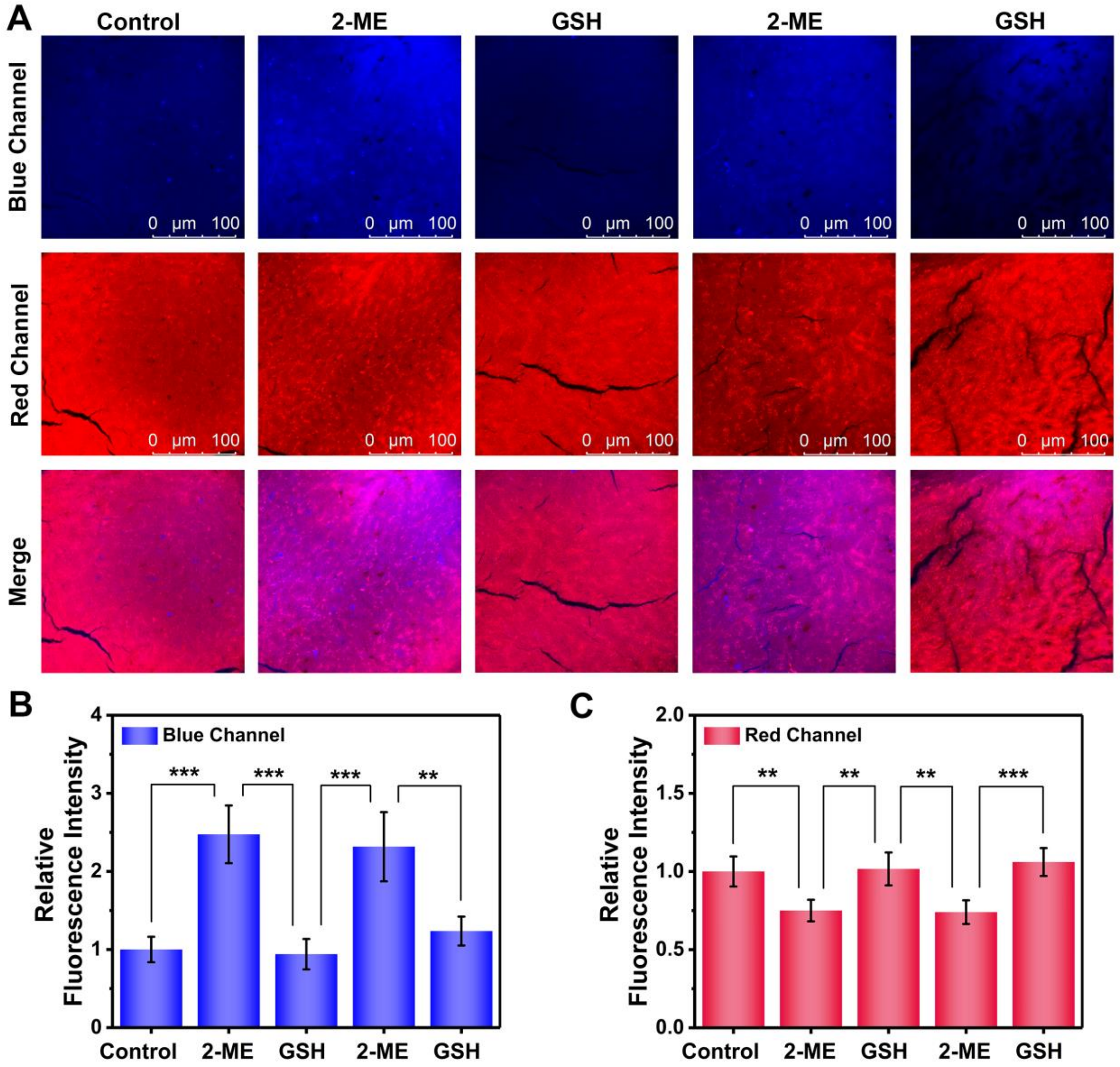

Figure S14. Reversible fluorescence imaging of $O_2^{\bullet-}$ and ATP fluctuations in mouse livers upon stimulation of 2-ME and GSH. (A) Reversible blue and red fluorescence images of $O_2^{\bullet-}$ (λ_{ex} = 405 nm, λ_{em} = 420-490 nm) and ATP (λ_{ex} = 514 nm, λ_{em} = 525-668 nm) in mouse livers by UDP injection (100 μM) upon intraperitoneal injection of 2-ME (15 μg/mL) and GSH (5 mM). (B), (C) Relative blue and red fluorescence intensity output of (A). Note: The blue and red fluorescence intensity of control group was defined as 1.0. The data are expressed as the mean ± SD. ***P < 0.001. **P < 0.01. Similar results were obtained in five independent experiments.

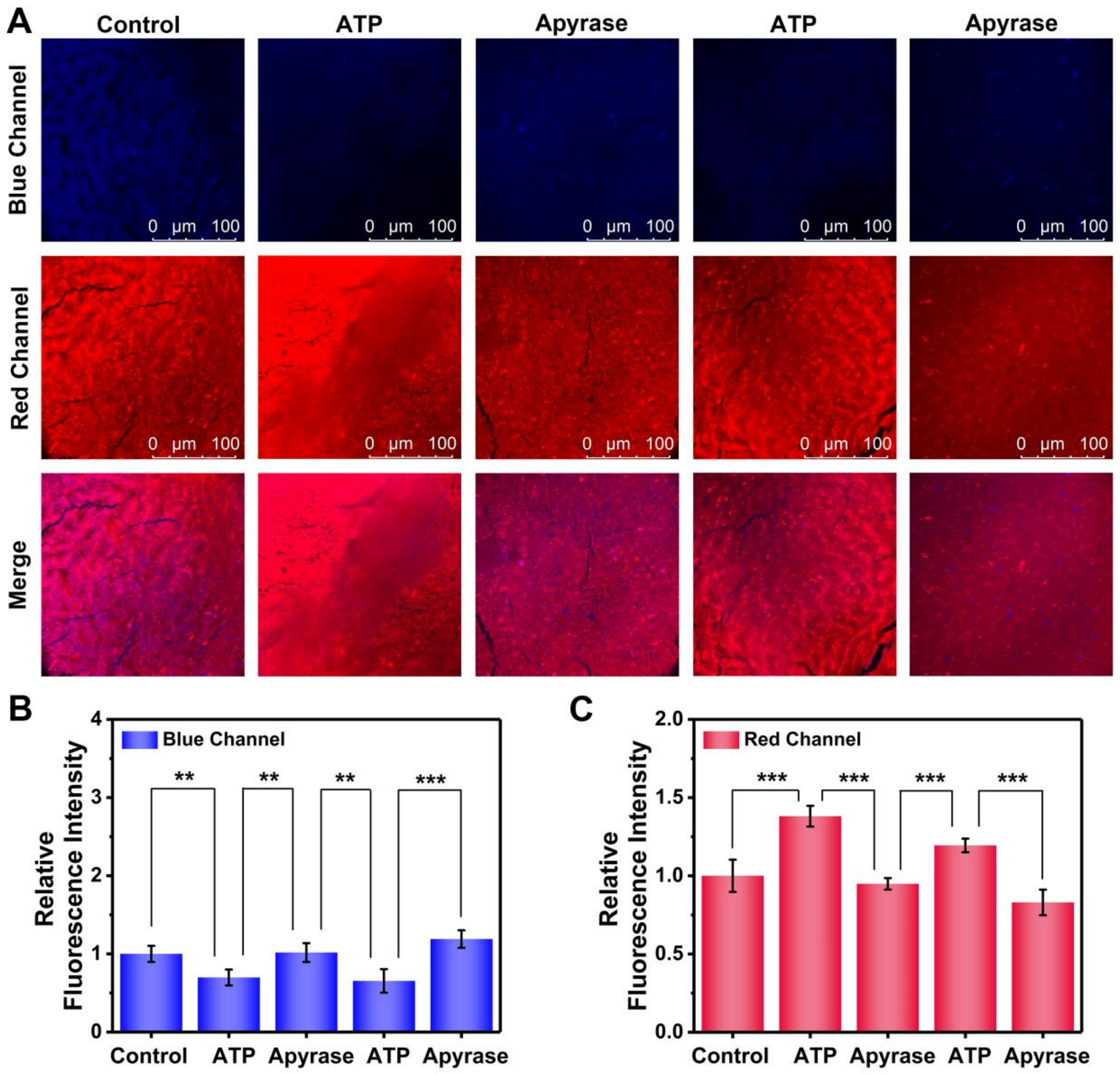

Figure S15. Reversible fluorescence imaging of $O_2^{\bullet-}$ and ATP fluctuations in mouse livers upon stimulation of ATP and apyrase. (A) Reversible blue and red fluorescence images of $O_2^{\bullet-}$ (λ_{ex} = 405 nm, λ_{em} = 420-490 nm) and ATP (λ_{ex} = 514 nm, λ_{em} = 525-668 nm) in mouse livers by UDP injection (100 μM) upon intraperitoneal injection of ATP (30 mM) and apyrase (3 U/N). (B), (C) Relative blue and red fluorescence intensity output of (A). Note: The blue and red fluorescence intensity of control group was defined as 1.0. The data are expressed as the mean ± SD. ***P < 0.001. **P < 0.01. Similar results were obtained in five independent experiments.

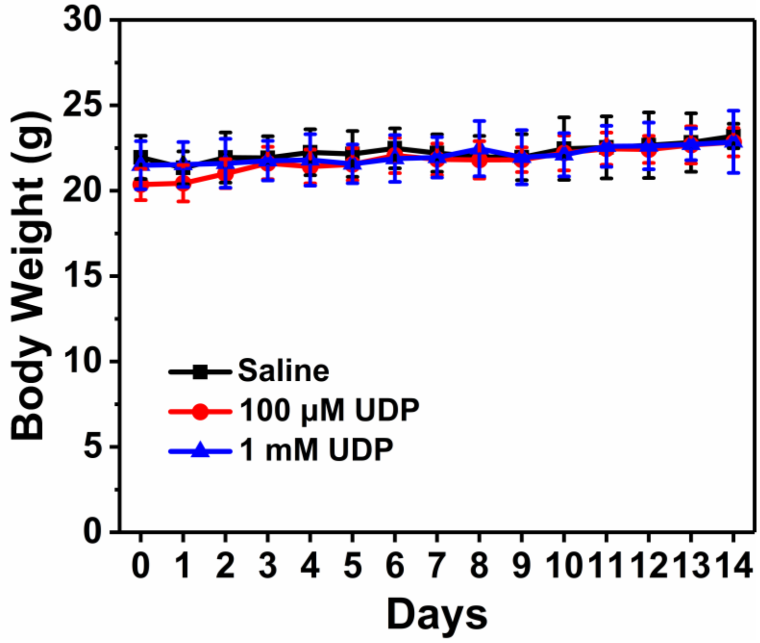

Figure S16. *In vivo* toxicity of UDP. Black line: The mice were intraperitoneally injected with 0.9 % NaCl aqueous solution (100 μL). Red line: The mice were intraperitoneally injected with 100 μM UDP (100 μL). Blue line: The mice were intraperitoneally injected with 1 mM UDP (100 μL).

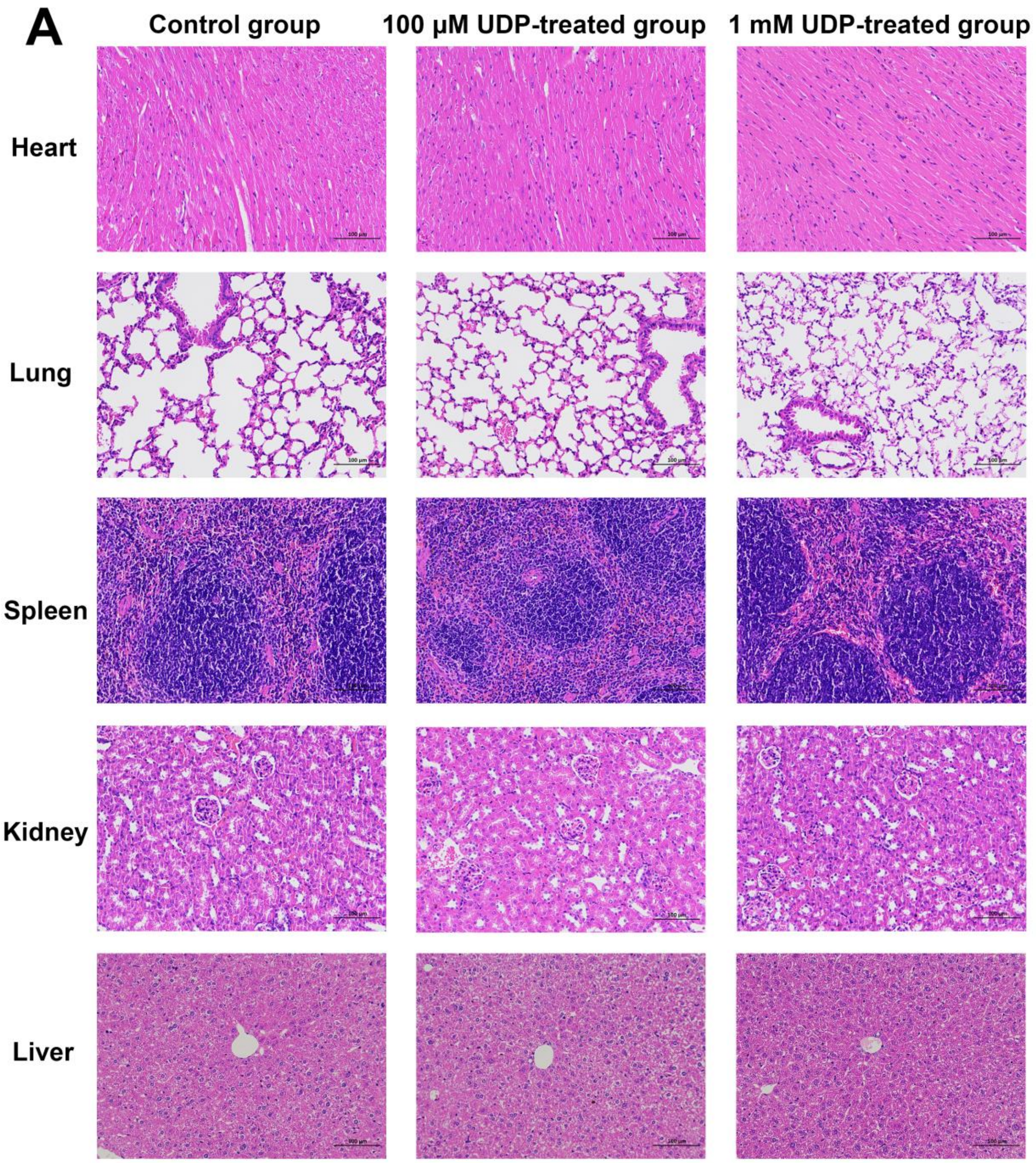

Figure S17. Hematoxylin and eosin (H&E) staining of major organ tissues (liver, spleen, lung, heart, and kidney) in control group, 100 μM UDP-treated group and 1 mM UDP-treated group. The control group were intraperitoneally injected with saline (0.9 % NaCl) solution every day for two weeks. The UDP-treated group were intraperitoneally administrated UDP for two weeks with experimental concentration of 100 μM or 1 mM.

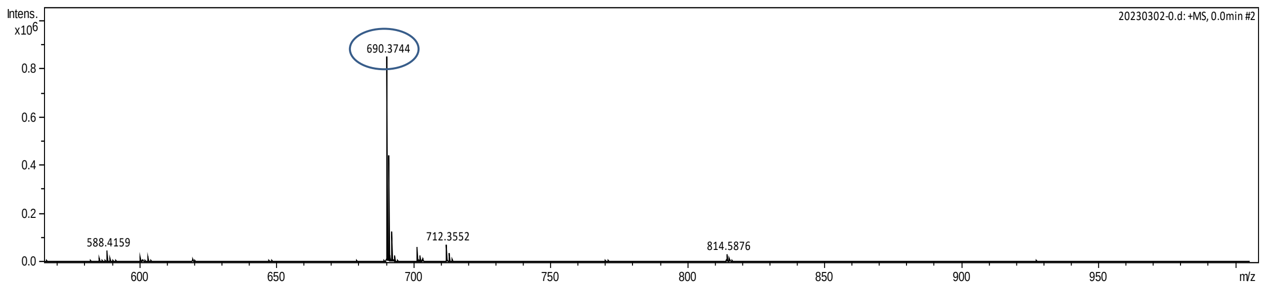

Figure S18. HRMS of UDP.

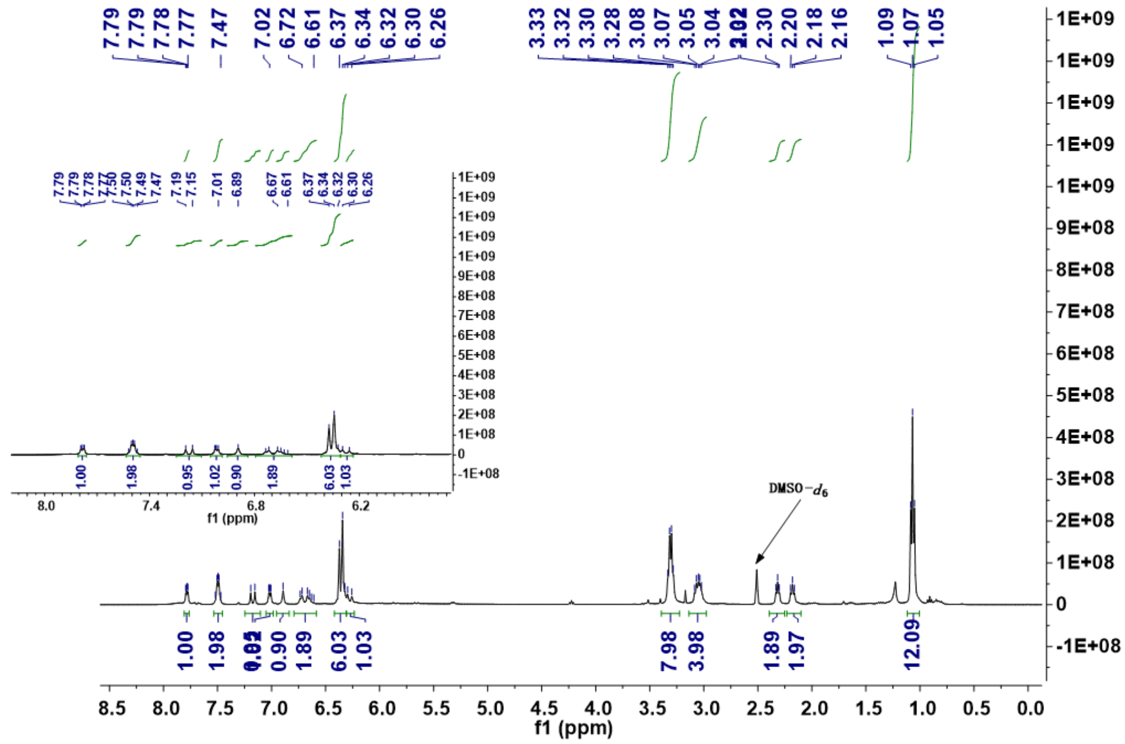

Figure S19. ^{1}H NMR (400 MHz, DMSO-d_6) of UDP.

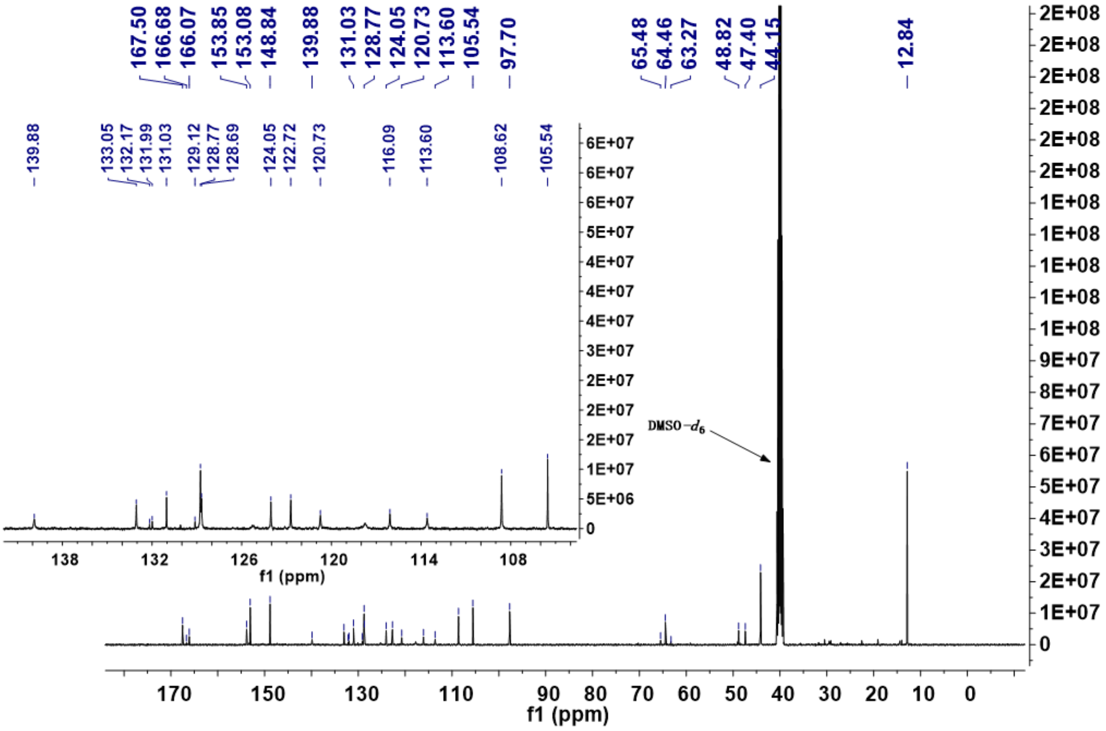

Figure S20. ^{13}C NMR (100 MHz, DMSO-d_6) of UDP.

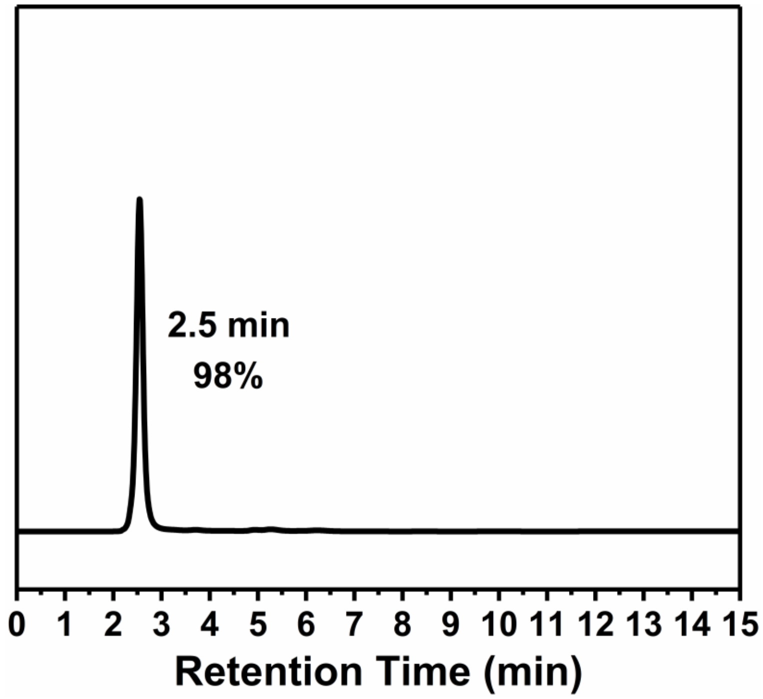

Figure S21. HPLC trace of UDP in 254 nm UV. HPLC analysis was carried out on a Shimadzu LC-16 system equipped with SPD-16 UV−vis detector. Deionized water was used as eluent A and methanol as eluent B. HPLC conditions: 14% of A, 86% of B. The injection volume was 10 μL. The parameters of the HPLC-MS analytical column used were C18-WR, 5 μm, 4.6 mm × 150 mm (GL Sciences). The flow rate is 0.7 mL/min.

Reference

[1] Wang, X.; Li, P.; Ding, Q.; Wu, C.; Zhang, W.; Tang, B. Illuminating the Function of the Hydroxyl Radical in the Brains of Mice with Depression Phenotypes by Two-Photon Fluorescence Imaging. *Angew. Chem., Int. Ed.* **2019**, *58*, 4674-4678.

[2] Singh, R. J.; Hogg, N.; Neese, F.; Joseph, J.; Kalyanaraman, B. TRAPPING OF NITRIC OXIDE FORMED DURING PHOTOLYSIS OF SODIUM NITROPRUSSIDE IN AQUEOUS AND LIPID PHASES: AN ELECTRON SPIN RESONANCE STUDY. *Photochem. Photobiol.* **1995**, *61*, 325-330.

[3] Feng, T.; Wan, J.; Li, P.; Ran, H.; Chen, H.; Wang, Z.; Zhang, L. A novel NIR-controlled NO release of sodium nitroprusside-doped Prussian blue nanoparticle for synergistic tumor treatment. *Biomaterials* **2019**, *214*, 119213.

[4] Yu, H.; Zhang, X.; Xiao, Y.; Zou, W.; Wang, L.; Jin, L. Targetable Fluorescent Probe for Monitoring Exogenous and Endogenous NO in Mitochondria of Living Cells. *Anal. Chem.* **2013**, *85*, 7076-7084.

[5] Dai, Z.; Tian, L.; Xiao, Y.; Ye, Z.; Zhang, R.; Yuan, J. A cell-membrane-permeable europium complex as an efficient luminescent probe for singlet oxygen. *J. Mater. Chem. B* **2013**, *1*, 924-927.

[6] Li, X.; Zhang, G.; Ma, H.; Zhang, D.; Li, J.; Zhu, D. 4,5-Dimethylthio-4‘-[2-(9-anthryloxy)ethylthio]tetrathiafulvalene, a Highly Selective and Sensitive Chemiluminescence Probe for Singlet Oxygen. *J. Am. Chem. Soc.* **2004**, *126*, 11543-11548.

[7] Kim, S.; Tachikawa, T.; Fujitsuka, M.; Majima, T. Far-Red Fluorescence Probe for Monitoring Singlet Oxygen during Photodynamic Therapy. *J. Am. Chem. Soc.* **2014**, *136*, 11707-11715.

[8] Chen, J.; Chen, L.; Wu, Y.; Fang, Y.; Zeng, F.; Wu, S.; Zhao, Y. A H_2O_2-activatable nanoprobe for diagnosing interstitial cystitis and liver ischemia-reperfusion injury via multispectral optoacoustic tomography and NIR-II fluorescent imaging. *Nat. Commun.* **2021**, *12*, 6870.
